# Transposable Element Expression in Acute Myeloid Leukemia Transcriptome and Prognosis

**DOI:** 10.1038/s41598-018-34189-x

**Published:** 2018-11-06

**Authors:** Anthony R. Colombo, Timothy Triche, Giridharan Ramsingh

**Affiliations:** 1 Jane Anne Nohl Division of Division of Hematology and Center for the Study of Blood Diseases, Keck School of Medicine of University of Southern California, Los Angeles, California 90033 USA; 2 Center for Epigenetics, Van Andel Research Institute, 333 Bostwick Ave NE, Grand Rapids, MI 49503 USA

## Abstract

Over half of the human genome is comprised of transposable elements (TE). Despite large-scale studies of the transcriptome in cancer, a comprehensive look at TE expression and its relationship to various mutations or prognosis has not been performed. We characterized the expression of TE in 178 adult acute myeloid leukemia (AML) patients using transcriptome data from The Cancer Genome Atlas. We characterized mutation specific dysregulation of TE expression using a multivariate linear model. We identified distinct patterns of TE expression associated with specific mutations and transcriptional networks. Genes regulating methylation was not associated with significant change in TE expression. Using an unpenalized cox regression analysis we identified a TE expression signature that predicted prognosis in AML. We identified 14 candidate prognostic TE transcripts (TEP) that classified AML as high/low-risk and this was independent of mutation-based and coding-gene expression based risk-stratification. TEP was able to predict prognosis in independent cohorts of 284 pediatric AML patients and 19 relapsed adult AML patients. This first comprehensive study of TE expression in AML demonstrates that TE expression can serve as a biomarker for prognosis in AML, and provides novel insights into the biology of AML. Studies characterizing its role in other cancers are warranted.

## Introduction

Approximately 50% of the genome is comprised of transposable elements (TE)^1^. Despite large-scale studies of the genome and transcriptome, the importance of TE in health has not been a focus of intense research until recently. TE have been implicated in cancer pathogenesis, but studies have mostly focused on their deleterious effects^2–6^. Very recently, beneficial roles of TE have been described. Induction of their expression leads to the activation of the viral recognition pathway and cancer cell death^7,8^. In addition, TE have been shown to regulate coding gene function^9–12^. Hence, they may indirectly alter the transcriptional networks to promote or inhibit cancer cell growth. This suggests that TE have complex and diverse functions in cancer, which has largely remained unexplored.

Prediction of prognosis using coding gene expression in cancer has been widely studied, resulting in development of many clinical assays. The role of TE in cancer prognosis has not been explored comprehensively. Hypomethylation of LINE1 elements in the cancer genome has been associated with prognosis^13^, however the role of TE expression in predicting prognosis in cancer is not well known.

Regulation of TE expression remains poorly understood. Like coding genes, TE can be regulated both transcriptionally and post-transcriptionally^14–17^. The role of coding gene mutations in altering gene regulatory networks is well known^18^, providing valuable information on the regulation of coding genes. However, how mutations affect transcription of TE has not been well characterized. By understanding the changes in TE expression with respect to specific mutations and chromosomal alterations in cancer, we can gain novel insight into how TE expression is regulated by the genes that are mutated in cancer.

In this study, we performed comprehensive analyses of the expression of TE in acute myeloid leukemia (AML). We analyzed mutation specific alterations in TE expression and correlated their expression pattern to transcriptional networks. We identified a TE expression signature that predicts prognosis in AML, paving the way for the development of novel prognostic TE biomarkers in AML.

## Methods

Please see the Supplementary Methods for details on the methodology.

## Results

### Mutation specific dysregulation of TE expression in AML

Mutations in AML are associated with distinct alterations in the expression of coding genes^19^, providing novel insight into gene regulation. In order to investigate the effect of mutations on TE expression, we investigated the relationship between specific mutations and expression of TE in AML.

**Figure 1:**
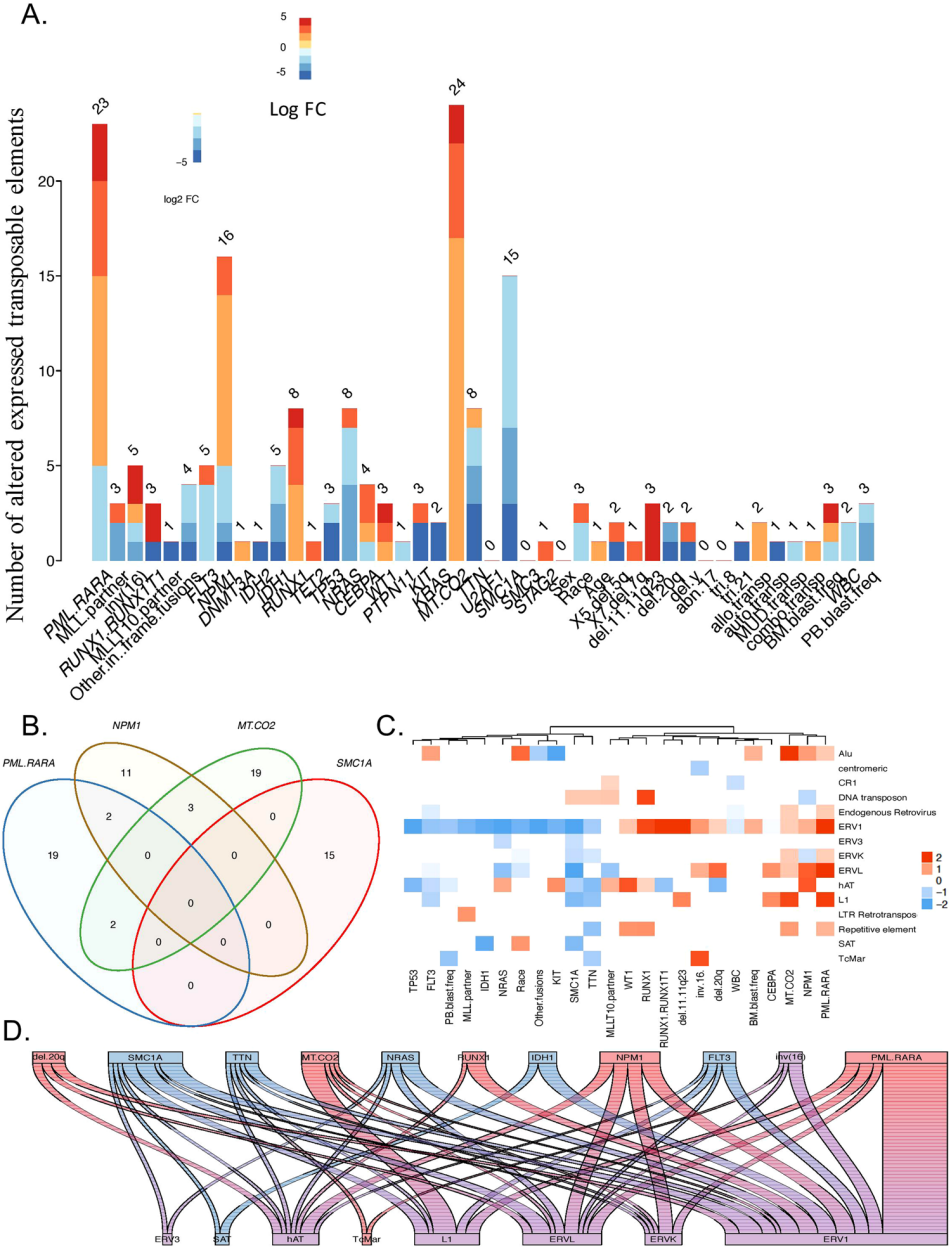
Mutation specific alteration in expression of transposable elements. (**A**) *The number of TE altered in expression (AE-TE) with respect to cytogenetics, mutations, and clinical factors* (Hierarchical test FDR ≤0.05; BH adjusted; n = 178). (**B**) *Venn diagram of AE-TE transcripts with respect to PML-RARα, MT.CO2, NPM1, SMC1A mutations*. Identifies significant AE-TE that are disjoint or shared among the 4 mutations/chromosomal alterations with the highest number of TE dysregulated. (**C**) *TE Biotype average significant dysregulation rates*. The biotype average dysregulation rates of statistically significant TE transcript identified from multiple regression per covariate (hierarchical test FDR ≤0.05; BH adjusted; n = 178). The y-axis are the biotypes, the x-axis are the model features. (**D**) *Altered expression summary of mutations/cytogenetics (top axis) corresponding to significant TE transcripts per biotype (bottom axis)*. Red is estimated up-regulated, and blue is estimated down-regulated; purple indicates an even mixture of both up and down regulated transcripts. The summary values considered only significant altered expression determined from multiple regression (hierarchical test FDR ≤0.05; BH adjusted; n = 178).

We analyzed the transcriptome of 178 AML patients from the Cancer Genome Atlas (TCGA) project using *Arkas*^20^, an RNA sequence analysis pipeline using detailed annotation information for TE and ENSEMBL non-TE (non-TE) transcripts. We used the multivariate empirical Bayesian linear model to comprehensively study the effect of various mutations on the expression of TE^19^. Chromosomal translocation *PML-RARα* was associated with the most number (23) of altered expression of TE transcripts (AE-TE), with most showing upregulation (Hierarchical FDR filtering, q ≤ 0.05) (Fig. 1A). This was followed by *MTCO2*, *NPM1*, *FLT3* and *RUNX1* mutations. Interestingly, the TE transcripts dysregulated with respect to the mutations associated with the most number of AE-TEs showed minimal overlap (Fig. 1B). Although CpG methylation has been shown to regulate the expression of TE^7,8,16^, *DNMT3A* and *TET2* mutations instead showed some of the lowest number of AE-TE.

TE biotypes exhibited specific alteration patterns with respect to mutational status (Fig. 1C,D). The TE transcripts within most TE biotypes exhibited a mix of both upregulation and down regulation with respect to mutations and chromosomal alterations, suggesting diversity in the regulation of TE transcripts within TE biotypes. *PML-RARα, MTC02* and *NPM1* were mostly associated with upregulation of TE, whereas *FLT3, IDH1* and *NRAS* were associated predominantly with downregulation of TE.

These findings suggest that the common mutations in AML are associated with a distinct pattern of alteration in TE expression.

### Correlating transcriptional networks with TE expression

**Figure 2:**
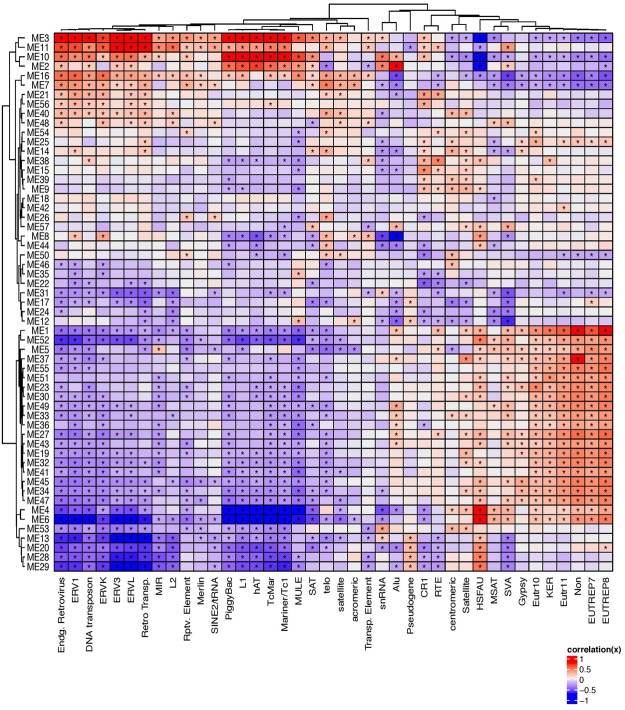
Correlating the transcript network with the expression of TE. Y-axis represents transcript ‘modules’ constructed by identifying non-TE transcripts based on the co-expression patterns. The X-axis denotes canonical TE biotypes used for correlating them. The centre figure represents the correlation matrix for the normalized gene ‘module’ expression and the TE type. *Indicates significant associations (pearson correlation p. value ≤ 0.05). The panel to the left of the Y-axis depicts significant (hierarchical test FDR <0.05; BH adjusted; n = 178) average dysregulation of the non-TE transcripts corresponding to each network module. For each module (y-axis), the Supplementary Table 1 depicts the corresponding non-TE module enrichment identifying the possible biological function.

TE are key regulators of coding-gene expression^1,9,10,12,21^. In order to gain insight into this in AML, we correlated the expression of TE biotypes with transcript networks. For this, the similarly expressed non-TE transcripts were grouped together, forming transcript modules as previously described by us^22^ (Y-axis in Fig. 2, Supplementary Table 1). The genes in the modules were likely to be co-regulated and/or functionally related^22^ (Supplementary Table 1). The modules were then correlated with the expression of specific TE biotypes (X-axis in Fig. 2, Supplementary Table 1). We observed that the TE biotypes formed distinct clusters based on its association with non-TE transcript modules, indicating diversity among TE biotypes.

We then summarized the network by averaging each module’s mutation/chromosomal specific alterations (Supplemental Fig. 2). Significantly altered expression of non-TE transcripts for each module with respect to covariates such as mutations/chromosomal alteration and demographics were measured (Supplementary Figure 1). Interestingly, *PML-RARα* was associated with the most (4428) alterations in the expression of non-TE transcripts (Supplementary Figure 1). The module alteration averages were clustered indicating dysregulation similarity between modules (Supplementary Figure 2). *PML-RARα* and *RUNX1/RUNX1T1* had similar module dysregulation averages (Supplementary Figure 2). This correlation matrix provided detailed information on the association between mutations, transcript networks, and the expression of various TE biotypes in AML.

### Prediction of prognosis using TE expression in AML

Expression of coding genes and non-coding genes such as microRNAs has been shown to predict the survival of many cancers including AML^23,24^. We investigated whether expression of TE can similarly predict prognosis in AML and if so, which TE are associated with good and bad prognosis.

**Figure 3:**
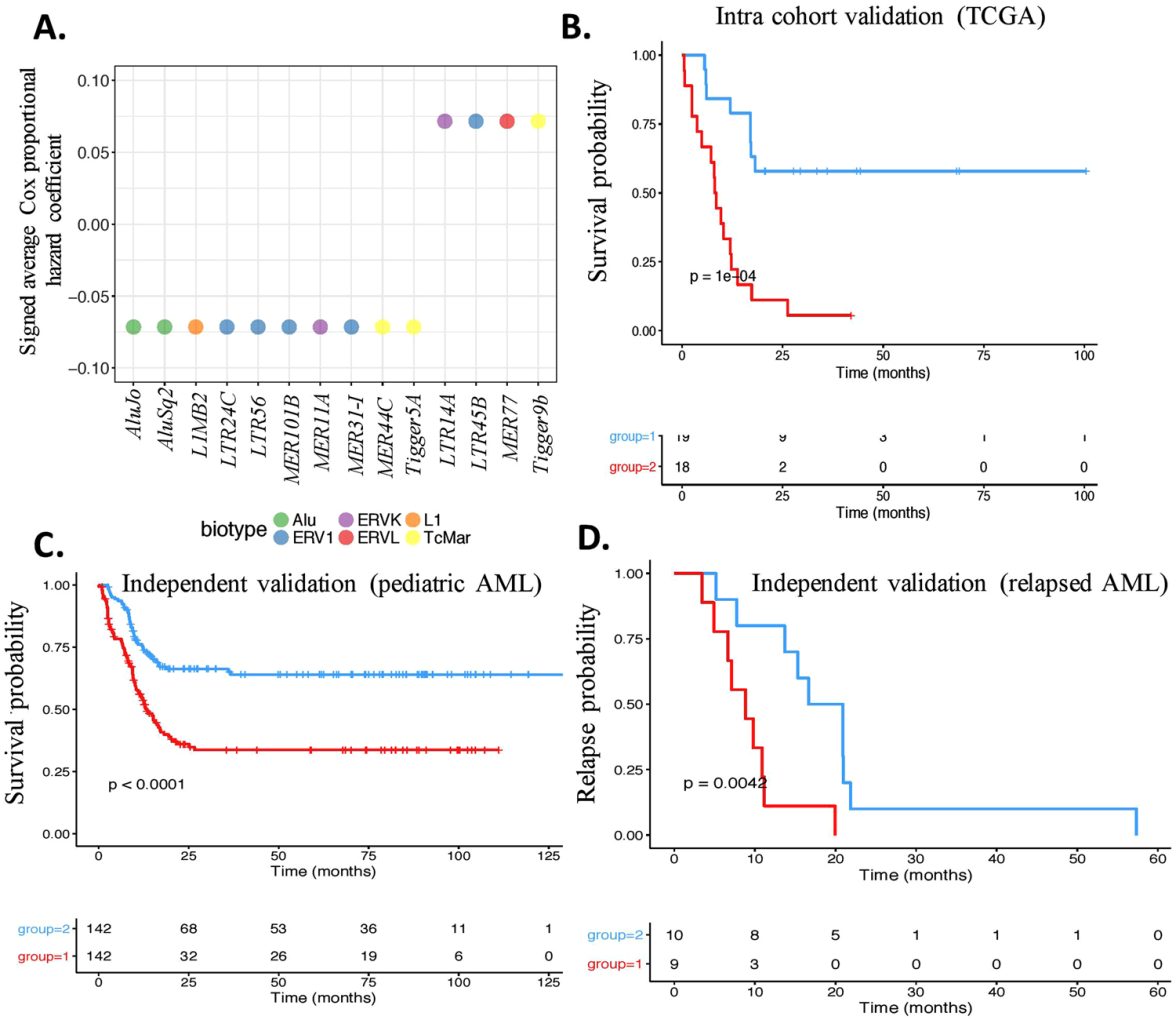
Kaplan-Meier survival plot using TEP expression in AML. (**A**) *TE transcripts identified to predict prognosis in AML (TEP)*. The x-axis depicts the candidate TEP. The y-axis depicts the signed average of the estimated hazard coefficients from Cox proportional hazards model. (**B**) *Intra-cohort validation of TEP in TCGA using a test cohort (N *= *37)*. Blue is favorable risk, and red is unfavorable risk. The y-axis is survival probability and the x-axis is time in months. The 2 patient risk classification groups were identified from TEP (log-rank-test, score log-rank-test, and Wald test p. value $\le$ 0.05). (**C**) *Independent validation of the TEP in pediatric AML using TARGET*. The y-axis is survival probability, and the x-axis is time in months. (**D**) *Independent validation of TEP in 19 relapsed AML patient samples*. The y-axis is relapse probability, and the x-axis is time in months. The 2 risk classification groups were determined from TEP (log-rank-test, score log-rank-test, and Wald test p. value <<0.05).

We identified candidate TE prognosticators (TEP) through nested sampling in TCGA. The nested training cohort was randomly selected on an approximately 60% intra-training and 40% intra-testing split. The training candidates were consistently prognostic TE transcripts and were selected based on an unpenalized univariate Cox proportional hazard model with a significance threshold of 0.0125 and 3-fold cross validation19. The training candidates were validated in the corresponding intra-test cohort using a Kaplan-Meir survival plot (Wald test q.value < 0.05). This identified 14 TE associated with prognosis (TEP) (Fig. 3A). We randomly selected 37 (20%) subjects into the intra validation test cohort and identified the hazard estimates of the 14 TEP using a multivariate Cox proportional hazard model. The estimated hazards of the 14 TEP were able to statistically distinguished patients with good and poor prognosis (Fig. 3B, Wald test = 3.62e-4, N = 37) (Supplementary Figure 3). A 3-fold cross validation yielded a mean correspondence index (c-index)^25–27^ of 0.6156. We examined the effects of age, gender, and ethnicity on TEP expression by comparing the Cox models which included demographical information and 14 TEP to the nested Cox model with 14 TEP covariates. Age, gender, and ethnicity did not significantly affect the 14 TEP in the intra-validation cohort (p. value = 0.12, Supplementary Table 2).

**Figure 4:**
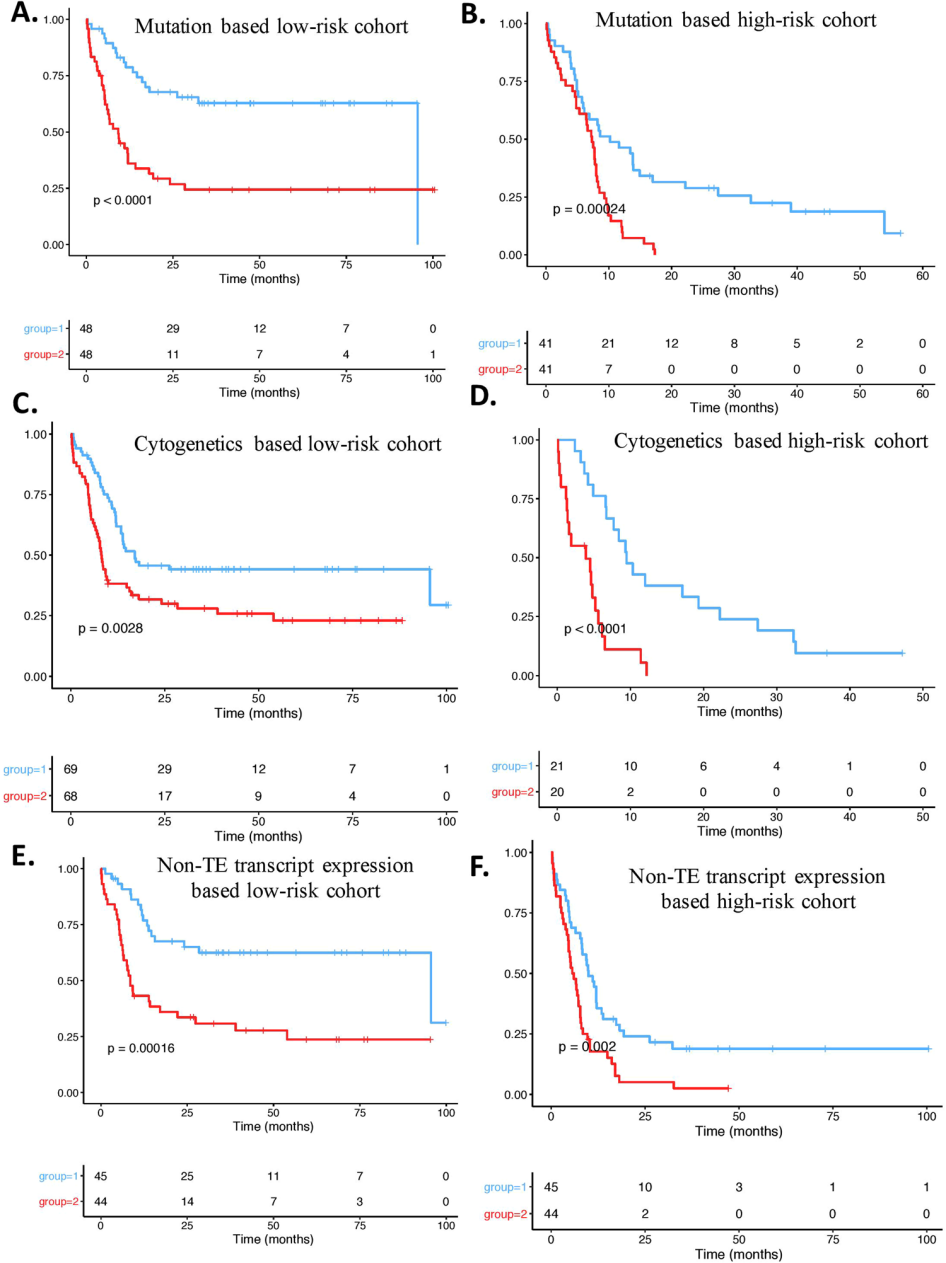
Utility of TEP expression in improving mutation based and coding gene expression based risk prediction. (**A**) *Improvement in prognostication of mutation based low-risk cohort (N* = *96)*. The x-axis is time in months, and the y-axis is survival probability. Kaplan-Meier sub-stratification of mutation based low risk patients using TEP (**B**) *Improvement in prognostication of mutation based high-risk group (N* = *82)*. Similar to (**A**). (**C**) *Improvement in prognostication of cytogenetics based low-risk group (N* = *137)*. Similar to (**A**). Low risk patients initially identified by using cytogenetics were sub-stratified using TEP. (**D**) *Improvement in prognostication of cytogenetics based high-risk group (N* = *41)*. High-risk patients initially identified by using cytogenetics were sub-stratified using TEP. (**E**) *Improvement in prognostication of nonTE transcript expression based low-risk group (N* = *99)*. Low risk patients initially identified from nonTE transcript expression were sub-stratified using TEP. (**D**) *Improvement in prognostication of non-TE transcript expression based high-risk group (N* = *99)*. High-risk patients initially identified from nonTE transcript expression were sub-stratified using TEP. Favorable risk is blue, and unfavorable risk identified in red (log-rank-test, score log-rank-test, and Wald test p. value < 0.025).

In order to further confirm the validity of the TEP in predicting prognosis in AML, we used 2 independent cohorts: 284 pediatric AML^28^ patients and 19 relapsed adult AML^29^ patients. Similar to identifying patient compounded risk scores in TCGA, we generated compound covariate summary values using the TEP expression and the corresponding Cox hazard estimates using pediatric AML patients. The TEP were able to stratify the 284 pediatric AML patients^28^ into favorable versus poor risk categories (Fig. 4C, Wald test p. value = 1.33e-06, N = 284) (Supplementary Figure 4). Age, gender and ethnicity did not significantly affect TEP (p. value = 0.4038, Supplementary Table 2).

The second independent validation in relapsed AML^29^ showed that the TEP stratified patients based on the differences in time to relapse between risk groups (Fig. 4D, Wald test p. value = 7.8–03, N = 19) (Supplementary Figure 4F). The age/gender adjusted hazard estimates for the 14 TEP were also examined in relapsed AML patients. Although for this smaller cohort the age/gender features did have an effect on the TEP covariates, the signed averages of the age/gender adjusted TEP hazard estimates were also prognostic (Wald test = 0.023, c-index = 0.646) (Supplementary Table 2). These results indicated the robustness of the discovery algorithm that predicted prognosis using TE expression in a large cancer cohort.

### TEP expression offers independent prognostic value

We then wanted to identify whether the TEP would independently improve the cytogenetics/mutation based and/or coding-gene expression based risk stratification. For this, we first used a Cox regression analysis and stratified the good-risk (N = 96) and poor-risk (N = 82) AML cohort using only the mutational signature. The TEP were then used to sub-stratify the low-risk (Fig. 4A) and high-risk groups (Fig. 4B) identified by mutational status. In the mutation based low-risk group TEP signature re-classified 48/96 patients to a higher-risk group (Fig. 4A, Kaplan-Meier Wald test p. value = 6.426e-05,3 fold cross-validated c-index = 0.58, N = 96). The TEP expression signature independently sub-stratified the mutational ‘higher-risk cohort’ into better-risk (41/82) groups (Fig. 4B, Kaplan-Meier Wald test p. value = 2.4e-04).

The TEP further classified cytogenetics and coding gene expression survival hazard categorizations. The 14 TEP identified 68 poorer risk patients from a pool of 137 good-risk patients identified based on cytogenetics (Fig. 4C, Kaplan-Meier Wald test p. value = 0.00284). And, the 14 TEP identified 21 better risk patients from a pool of 41 poor-risk patients identified by cytogenetics (Fig. 4D) (Kaplan-Meier Wald test p. value = 4.91e-05). Likewise, the TEP identified 45 poorer risk patients from a pool of 99 good-risk patients identified based on gene expression (Fig. 4E, KM Wald test p. value = 1.58e-04). The TEP identified 45 better risk patients from a pool of 99 poor-risk patients identified by gene expression (Fig. 4F) (KM Wald test p. value = 1.97e-03).

Overall, this indicated that the TEP could provide robust independent prognostic value and can improve the prognostic ability obtained by mutational status, cytogenetics or coding gene expression in AML.

### TEP associated coding gene expression and gene networks

**Figure 5:**
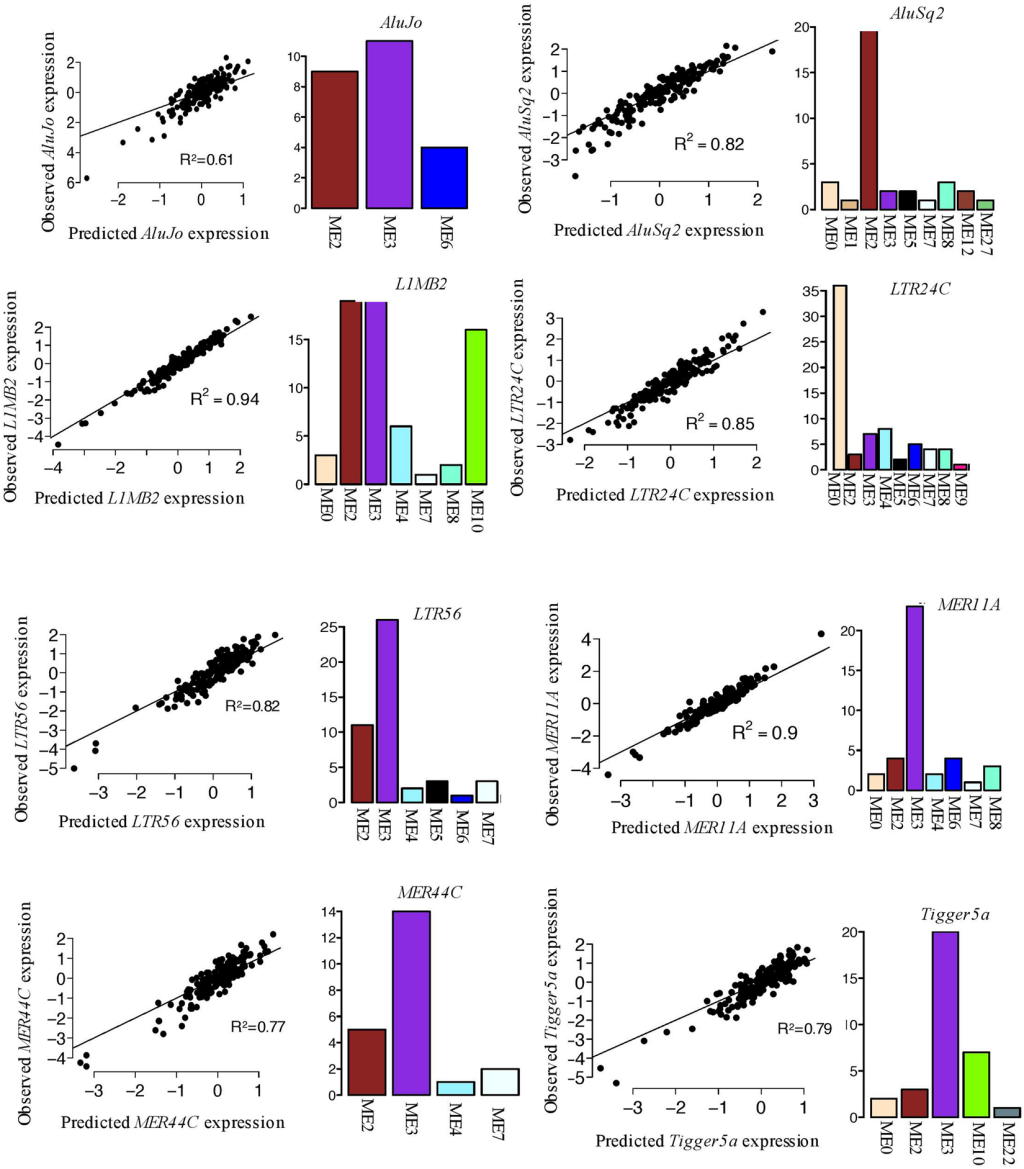
TEP associated coding gene expression and their corresponding network module memberships. For each TEP, independent coding gene expression predictors were identified using penalized regression. The x-axis on the linear model plots depict the scaled predicted TEP expression determined by the selected coding genes, and the y-axis depict the scaled observed TEP expression. The adjacent bar plots show the gene network module membership distribution of the selected independent coding gene predictors from Fig. 2. The bar-plot x-axis show the module ID from Fig. 2, the y-axis is the corresponding frequency of gene predictors residing in the given module.

TEs constitute diverse transcripts that fall within several TE biotypes. However, the functional roles of individual TE transcripts are yet to be characterized. In order to gain insight into the potential functional role of the TEPs in AML, we analyzed the coding genes that appear highly correlated in expression to TEPs. A majority of the coding genes (Supplementary Table 3), which are associated to TEPs such as *AluJo, L1MB2, LTR56, MER11A, MER44C*, and *Tigger5a*, were members of the network module 3 (ME3) (Figs 2 and 5). A functional pathway analysis of the coding genes in ME 3 using DAVID^30^, revealed that the genes in ME3 were enriched in biological processes such as regulation of transcription (GO:0006355, GO:0006351), detection of chemical stimulus involved in sensory perception of taste (GO: 0001580, GO:0050909), nucleic acid binding, metal ion binding, and ATP binding (GO:0003676, GO:0046872, GO:0005524 respectively).

### Risk-stratification of AML based on a combination of mutations and TEP expression

**Figure 6:**
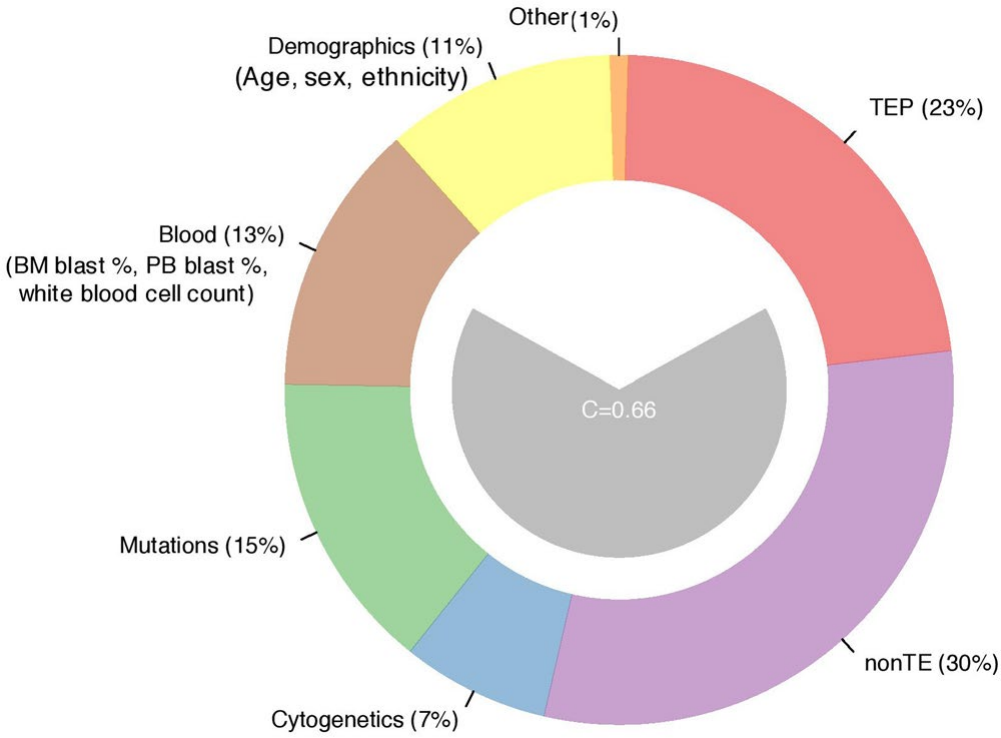
Prognostic contributions for multiple clinically relevant features. The center (grey) polygon depicts the C-statistic derived from a multivariate Cox proportional hazards model. The multiple predictive variables were TEP expression, nonTE expression, cytogenetics, mutations, blood, and demographic variables. The percentages indicate the overall group contribution to the C-statistic.

We developed a composite model combining the prognostic value of both the mutations, cytogenetics, non-TE expression and TEP expression to understand the relative effect of mutations and TEP in various mutational sub-categories of AML. We identified that non-TE transcript expression profile, TEP and mutational status were the primary contributors of risk prediction in adult AML (Fig. 6) (Supplementary Table 4).

**Figure 7:**
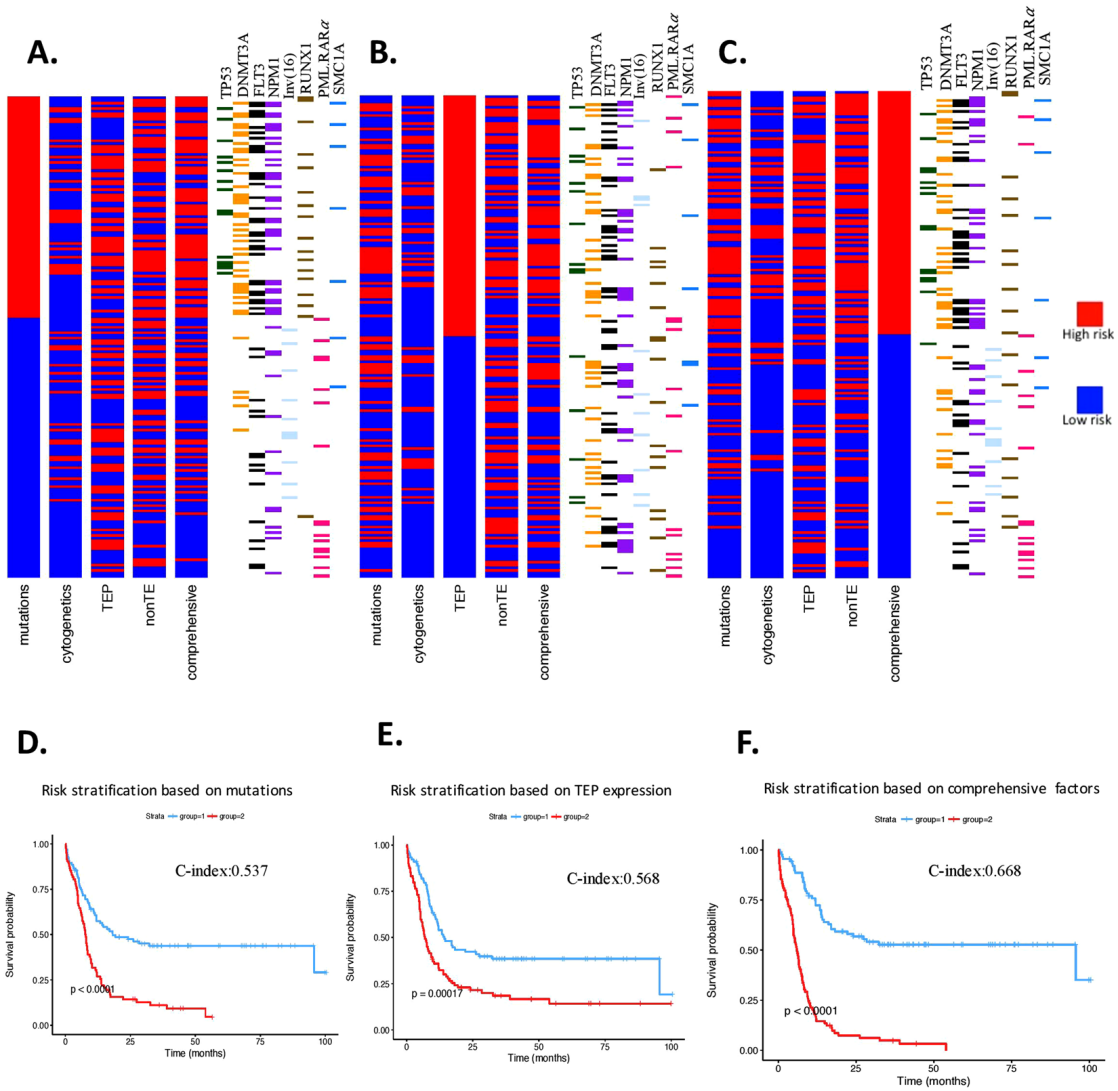
Comprehensive risk-stratification of AML. (**A**) *AML patient classified based on mutational status*. The mutation based risk classification for each patient is depicted on the left. Right-adjacent depicts clinically relevant mutations or cytogenetic annotations corresponding to the patient classification bar plot. Each bar plot (4) depicts independent risk classification models using covariates such as mutations, cytogenetics, 14 TEP, nonTE, or a comprehensive model including mutations, cytogenetics, nonTE, TEP and demographical information. High risk (red) and low risk (blue) represents patient’s risk classification. (**B**) *TEP based risk classification model*. The TEP based risk stratification for each patient and the corresponding clinical profile. (**C**) *The comprehensive classification model*. The patients are ordered from the risk classification determined by the comprehensive model utilizing mutational status, cytogenetics, TEP expression and nonTE expression. Each subject’s clinical profile is depicted to the right of the classification bar plots.

Using mutations alone the 178 AML patients were classified in to 96 low-risk and 82 high-risk patients. This risk stratification based placed most of *DNMT3A*, *TP53*, *RUNX1* and *FLT3* mutations into high-risk category and *NPM1* mutation, *PML-RARα* and inv (16) into low-risk category (Fig. 7A). We then re-classified all 178 adult AML patients using a combination of mutational status, cytogenetics, non-TE expression and TEP expression. The comprehensive model re-classified 48/96 mutation based low-risk and 41/82 mutation based high-risk patients to worse and better prognosis respectively (Fig. 4A/B). We analyzed the mutational categories of patients that were reclassified in these groups (Fig. 7B). In the mutation based high-risk group, the TEP model re-classified a large majority of patients with *DNMT3A*, *DNMT3A* plus *NPM1*, *FLT3* plus *NPM1*, and *RUNX1* mutations as low-risk. Only one of the *TP53-*mutated AML patients identified as high-risk from the mutational based model was reclassified as low-risk using TEP (Fig. 7A/B). This suggested that AML patients with *DNMT3A*, *RUNX1, NPM1* and *FLT3*, mutations constitute a diverse group with regards to their prognosis. These findings demonstrate that by incorporating the expression of the 14 TEP in a comprehensive model that includes mutational status, cytogenetics and non-TE expression pattern, we can improve the ability to predict prognosis in AML.

## Discussion

This is the first study to comprehensively characterize the expression of TE expression at the transcript level, demonstrating novel roles for TE in cancer. *PML-RARα* chromosomal translocation was identified to be associated with the most dysregulation of TE expression, with majority of the dysregulated TEs exhibiting upregulation of expression in AML. PML-RARα fusion protein has been shown to be a transcriptional repressor through its role regulating DNA methylation and chromatin modification^31^ Its specific role in regulating TEs has not been previously known. Whether treatment with ATRA (All-trans retinoic acid), or arsenic trioxide, in *PML-RARa* translocated AML leads to changes in TE expression and their role in the pathogenesis of *PML-RARa* translocated AML needs to be investigated.

Although methylation has been previously shown to regulate the expression of TE^7,8^, our study showed very little association between mutations in methylation regulating genes (*DNMT3A* and *TET2*) and dysregulation of TE expression. However, we found strong association between mutations in *NPM1, MTCO2, and SMC1A*. Interestingly *FLT3* mutation *was largely associated with* suppression of TE, and *IDH1*, and *TP53* were the only mutational features showing complete uniform suppression. Studies investigating the mechanism of how these genes regulate TE expression will be needed. Recent report suggested that *TP53*-mutated AML are highly susceptible to treatment with hypomethylating agents^32^, which have been shown to activate the expression of TE and the downstream interferon cascade, leading to cancer cell death^7,8,33^. The mechanism high responsive rate to hypomethylating agents in *TP53*-mutated AML is not completely characterized. Our study does not show a high level of dysregulation of TE in *TP53*-mutated AML, but the dysregulated TEs were all downregulated. The relation of this finding to the mechanism of action of hypomethylating agents in *TP53*-mutated AML needs to be investigated.

TE constitutes a diverse group of transcripts, which likely have diverse function, as demonstrated by our network analysis. Interestingly, *AluJo, AluSq2, L1MB2, LTR24C, LTR56, MER101B, MER11A, MER31-I, MER44C*, and *Tigger5a* transcripts were associated with low hazards and *LTR14A, LTR45B, MER77* and *Tigger9b* were classified as hazardous. Interestingly, *L1MB2*, a LINE-1 element, was calculated as low hazardous which contrasts other studies describing the re-activation of LINE-1 elements as associated with increased genomic instability and hence hazardous in cancer. This indicates that LINE-1 may play a diverse role of the development of cancer. Additionally, the other TE transcripts within the same class/type were associated with varying hazard estimates, further indicating diversity within TE. We predict that individual TE transcripts are as diverse as coding genes in their function. It is likely that some of the TE that have active transposition activity promoting tumorigenesis^6^, and others that have defective transposase activity performing other functions such as activating tumor immunogenicity via interferon activity. In this context, we recently showed that leukemic stem cells in AML, which are the most resilient to treatment, have suppressed TE expression^22^. A recent study also reported that TE is suppressed in chemotherapy resistant cancer cells^34^. These findings demonstrate that suppression of TE in cancer could possibly play a role in cancer evolution by protecting the cells from immune mediated cell death.

Further understanding disease pathogenesis requires a closer investigation of the regulation mechanisms of the 14 TEP transcripts. This study is limited in its ability to characterize short interspersed nuclear elements (SINE), since most of them do not contain polyA tail and that the sequencing libraries were generated using polyA selection method. The SINE TEs identified using our pipeline were a sub-group of SINEs that have polyA tail. Future large scale studies could include non-polyA selection protocols which would provide opportunities for examining SINEs in better detail.

Our comprehensive model suggests that the expression of 14 TEP transcripts contributes significantly to the prognostication. We propose an improved prognostic algorithm in AML utilizing a comprehensive model that includes mutational status, cytogenetic status, coding gene expression and TEP expression. The utility of TEP in risk stratifying AML needs to be further validated using orthogonal assays in future studies.

This is the first study demonstrating the utility of TE expression signature in predicting outcomes in cancer. This study establishes the analytical foundation to investigate the role of TE in other cancers. Whether the prognostic TEP identified in AML overlaps with other cancer needs to be investigated.

## Electronic supplementary material

Supplementary Figures, Tables, and methods

Supplementary data set
