# Supplementary material for: Transposable Element Expression in Acute Myeloid Leukemia Transcriptome and Prognosis
Source: Sci Rep. 2018 Nov 6;8:16449. doi: 10.1038/s41598-018-34189-x (PMC6219593; doi:10.1038/s41598-018-34189-x)
Supplement: Supplementary file 1 — Supplementary Figures, Tables, and methods [file 41598_2018_34189_MOESM1_ESM.pdf]

Transposable Element Expression in Acute Myeloid Leukemia Transcriptome and Prognosis.

Anthony R. Colombo, Timothy Triche Jr., and Giridharan Ramsingh.

Supplementary Files

Supplementary figure 1.

A.

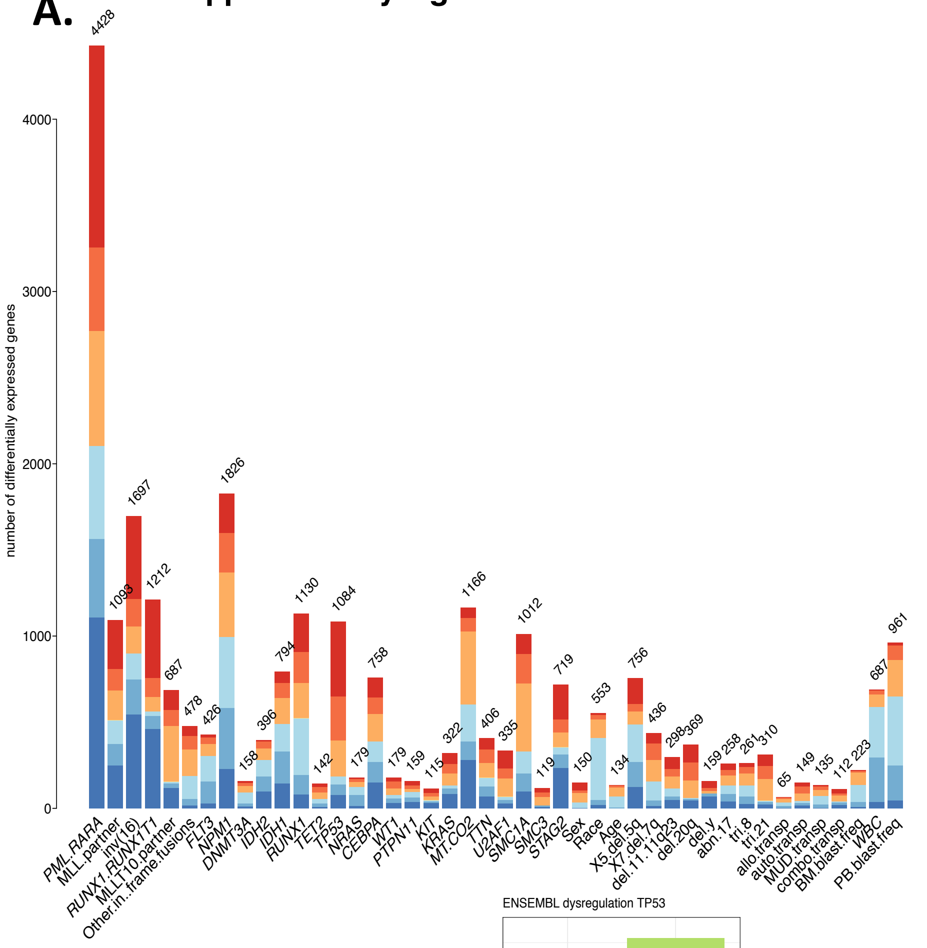

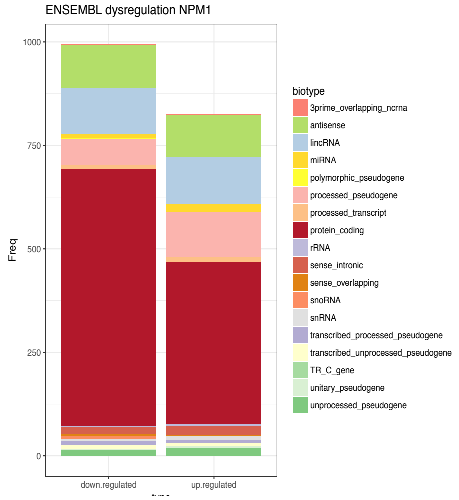

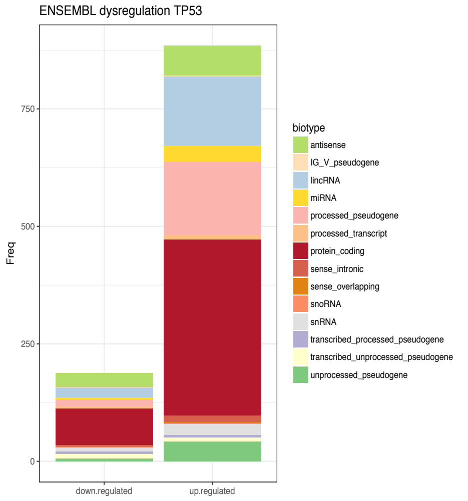

A) Depicts significantly altered expression of nonTE (hierarchical test, FDR BH <0.05) corresponding to multiple features.

B) The bar plots depict the total significantly altered nonTE dysregulated for various predictive features. The x-axis depicts the group (down-regulated or up-regulated). The y-axis depicts the frequency of significantly altered elements corresponding to the ENSEMBL biotype class.

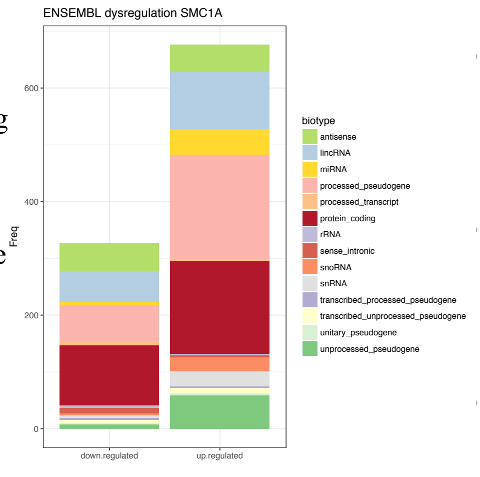

B.

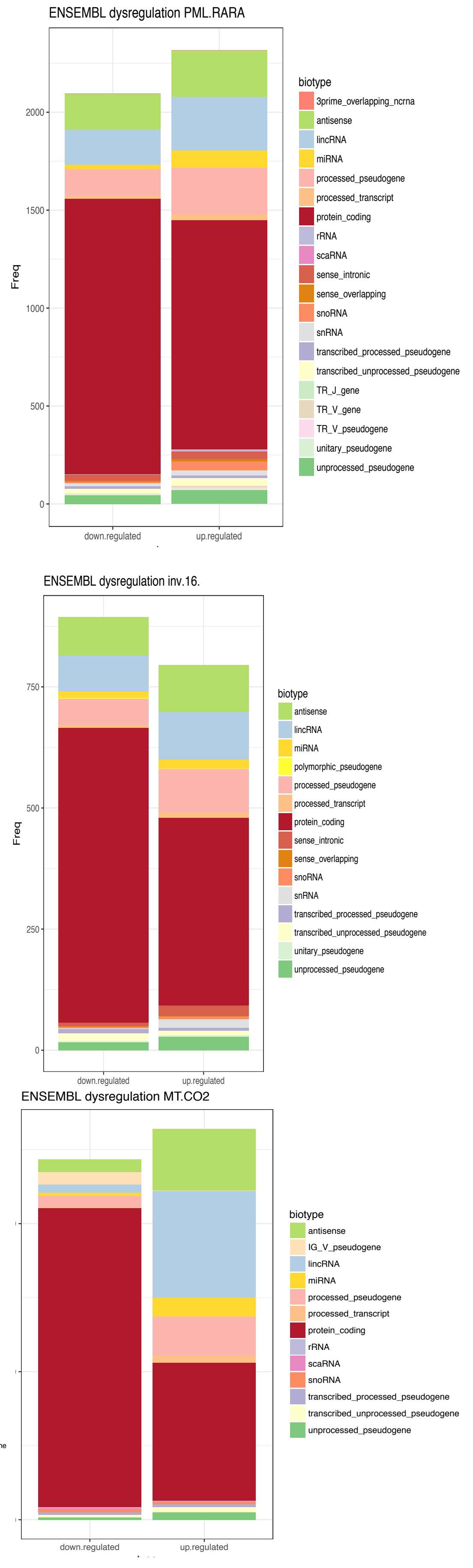

Supplementary figure 2.

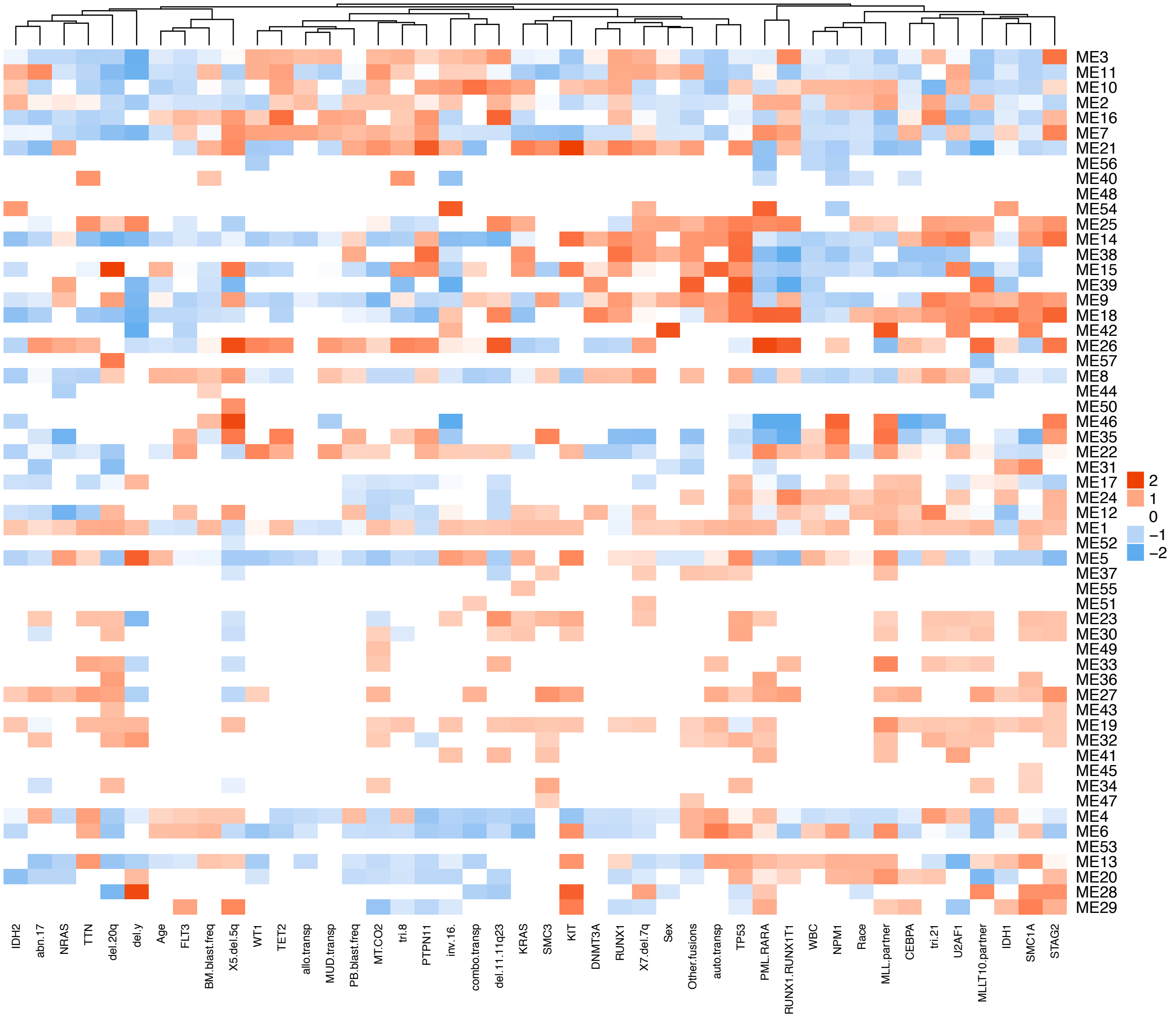

Module average dysregulation for multiple predictive features. The x-axis depicts the predictive features. The y-axis depicts the modules constructed by network analysis. The module dysregulation average was summarized from the significant altered non-TE, predicted by the multiple regression (supplement figure 1), that corresponded to the given module.

Supplementary figure 3.

A.

	coef	exp(coef)	se(coef)	z	Pr(>|z|)
wald.test	1.57896635	4.84994009	0.44273068	3.56642629	0.00036188

A. Wald test statistics from the Kaplan-Meier survival curve of the TCGA intra-validation cohort of 37 subjects. Wald test on the 2 risk groups identified by TEP. The subjects were assigned into 2 risk groups after compounding Cox proportional hazard estimates with the corresponding TEP expression. The concordance measure using the intra-validation subjects and 14 TEP was 0.6156. The intra cohort validation subjects were identified by performing an 80/20 split of TCGA subjects.

B.

TEP	Signed average of hazard coefficients	Biotype
AluJo	-0.071428571	Alu
AluSq2	-0.071428571	Alu
L1MB2	-0.071428571	L1
LTR14A	0.071428571	ERVK
LTR24C	-0.071428571	ERV1
LTR45B	0.071428571	ERV1
LTR56	-0.071428571	ERV1
MER101B	-0.071428571	ERV1
MER11A	-0.071428571	ERVK
MER31_I	-0.071428571	ERV1
MER44C	-0.071428571	TcMar
MER77	0.071428571	ERVL
TIGGER5A	-0.071428571	TcMar
Tigger9b	0.071428571	TcMar

B. *Signed averages of Cox proportional hazard estimates of TEP*. Signed averages of TEP hazard estimates which include the TEP biotype family.

C.

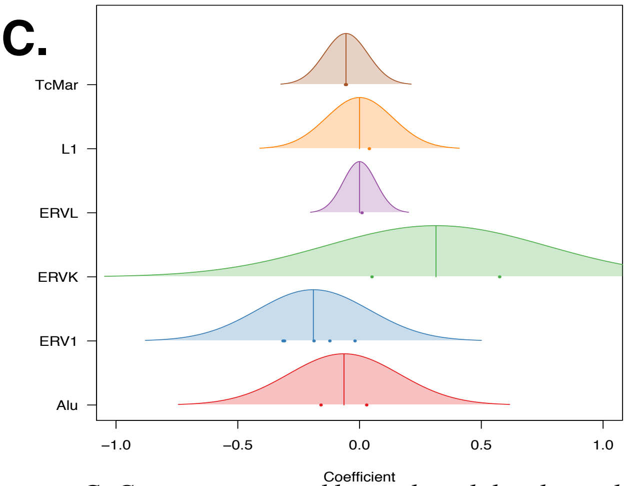

C. Cox proportional hazard model with random effects of TEP biotype families. The x-axis is the estimated biotype hazard coefficient. The y-axis is the TEP biotypes.

D.

signed averaging	coef	exp(coef)	se(coef)	z	Pr(>|z|)
wald.test	0.97811019	2.65942569	0.48381876	2.02164587	0.04321295

D. *Kaplan-Meier survival analysis using signed averages Wald test statistic.* The signed averages of hazard coefficients upon compounding into patient risk scores tends to be conservative, thus balancing the increased power obtained from experiments with large sample sizes. The corresponding Kaplan-Meier plot is depicted in ***F.***

E.

cross-fold validation	Harrel's c-index
1	0.744444444
2	0.580952381
3	0.693877551
4	0.527777778
5	0.666666667
average C-index across folds	0.642743764

E. *Concordance index using 5-fold cross validation of all TCGA subjects.* The 5-fold cross validation concordance measure for each random fold of all TCGA subjects (N=178). The 14 TEP expression were used as the survival predictors.

F.

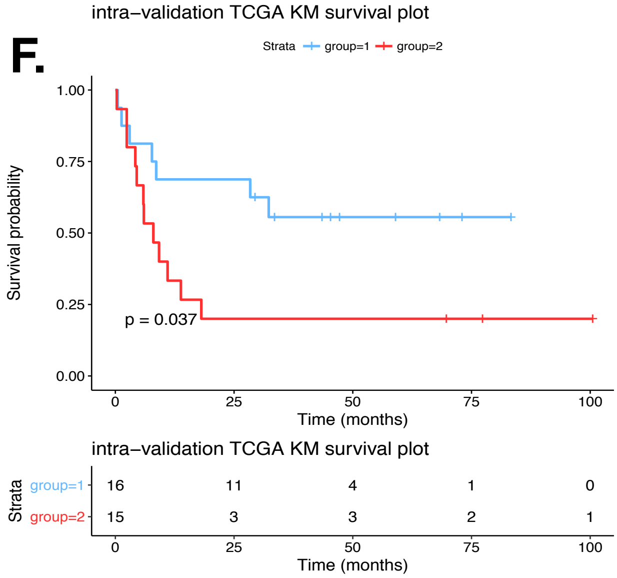

F. *Intra cohort validation Kaplan-Meier survival plot derived from signed averages of TEP hazard estimates.*

Supplementary figure 4.

A.

TARGET	coef	exp(coef)	se(coef)	z	Pr(>|z|)
wald.test	0.88557082	2.42436788	0.18314191	4.83543511	1.33E-06

A. *Wald test statistics from the Kaplan-Meier survival curve of the pediatric AML subjects (N=284).* The subjects were assigned into 2 risk groups after compounding Cox PH estimates with the corresponding TEP expression.

B.

TEP	Signed averages of hazard coefficients	Biotype
AluJo	0.071428571	Alu
AluSq2	-0.071428571	Alu
L1MB2	0.071428571	L1
LTR14A	-0.071428571	ERVK
LTR24C	-0.071428571	ERV1
LTR45B	0.071428571	ERV1
LTR56	0.071428571	ERV1
MER101B	-0.071428571	ERV1
MER11A	-0.071428571	ERVK
MER31_I	-0.071428571	ERV1
MER44C	-0.071428571	TcMar
MER77	-0.071428571	ERVL
TIGGER5A	0.071428571	TcMar
Tigger9b	0.071428571	TcMar

C.

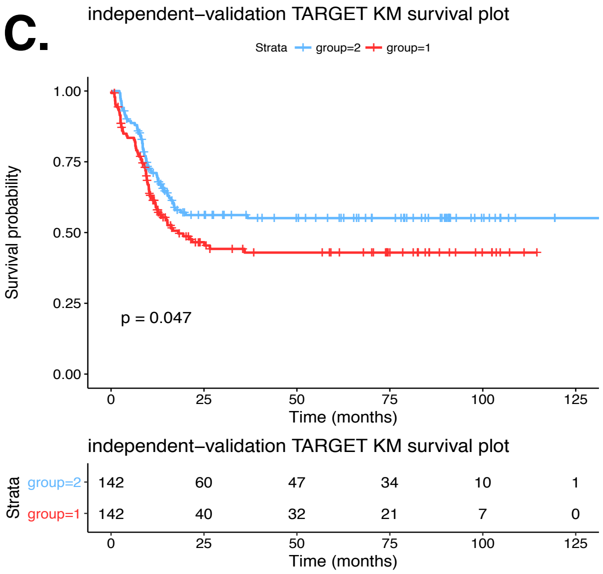

B. *Signed averages of Cox proportional hazard estimates of TEP.* Signed averages of TEP (via pediatric AML) hazard estimates which include the TEP biotype family.

C. *Pediatric AML Kaplan-Meier survival plot of 2 risk classification groups derived using signed averages of Cox hazard estimates of 14 TEP.*

D.

igned averages of hazard coefficients	coef	exp(coef)	se(coef)	z	Pr(>lzl)
(TARGET) wald.test	0.348402	1.416801	0.17636	1.975509	0.04821

D. *Wald test statistic of risk classification derived from signed averages of Cox hazard estimates.* The statistic corresponds to ***C.***

E.

fold validation	c-index of fold
1	0.627787307
2	0.680602007
3	0.473262032
4	0.55942029
5	0.600749064
average C-index across folds	0.58836414

E*. Concordance C-statistic using 5-fold cross validation of all pediatric AML subjects.* The 5-fold cross validation concordance measure for each random fold of pediatric AML. The 14 TEP expression were used as the survival predictors. The average concordance measure was dependent of the random subject sampling assignment used in five-fold cross validation. The average concordance index ranged from 0.589-0.6102 using the 14 TEP in pediatric AML.

F.

MSKCC	coef	exp(coef)	se(coef)	z	Pr(>|z|)
wald.test	1.56370186	4.7764704	0.58841227	2.65749363	0.00787241

F. *Wald test statistics from the Kaplan-Meier relapse probability curve of the relapsed AML subjects (N=19).* The subjects were assigned into 2 risk groups after compounding Cox PH coefficients estimates with the 14 TEP expression. The concordance measure (3-fold cross-validation) was 0.602.

Supplementary table 1.

modules	term
ME3	bitter taste receptor activity,metal ion binding,cation binding
ME11	plasma membrane,sensory perception,detection of stimulus involved in sensory perception
ME10	centriole,heart development,establishment of RNA localization to telomere
ME2	substrate-specific transmembrane transporter activity,transmembrane transporter activity,ion transmembrane transporter activity
ME16	DNA binding,nucleic acid binding,regulation of nitrogen compound metabolic process
ME7	DNA binding,metal ion binding,regulation of transcription, DNA-templated
ME21	plasma membrane,cell periphery,thrombin-activated receptor signaling pathway
ME56	U4 snRNP,U2-type prespliceosome,U2 snRNP
ME40	carboxylic acid catabolic process,L-lysine catabolic process to acetyl-CoA via L-pipecolate,negative regulation of lens fiber cell differentiation
ME48	mitochondrial genome maintenance,reproduction,vacuole inheritance
ME54	type 2A serotonin receptor binding,chondrocyte development involved in endochondral bone morphogenesis,G-protein coupled serotonin receptor binding
ME25	neurotransmitter receptor activity,transmembrane signaling receptor activity,receptor activity
ME14	hemoglobin complex,oxygen transporter activity,oxygen transport
ME38	proteinaceous extracellular matrix,cell adhesion,extracellular matrix
ME15	T cell activation,immune response,immune system process
ME39	platelet activation,platelet alpha granule,platelet degranulation
ME9	cell-cell signaling,synapse organization,multicellular organism development
ME18	proteinaceous extracellular matrix,extracellular space,extracellular matrix
ME42	histone H3-K27 demethylation,histone demethylase activity (H3-K27 specific),sequestering of actin monomers
ME26	neurogenesis,nervous system development,generation of neurons
ME57	mitochondrial genome maintenance,reproduction,vacuole inheritance
ME8	nuclear lumen,nucleoplasm,nucleus
ME44	cell cycle,chromosome organization,DNA repair
ME50	no GO terms identified,no GO terms identified
ME46	embryonic skeletal system morphogenesis,anterior/posterior pattern specification,sequence-specific DNA binding
ME35	pattern specification process,kidney morphogenesis,embryonic skeletal system morphogenesis
ME22	cilium assembly,cilium organization,positive regulation of neutrophil apoptotic process
ME31	chiasma assembly,blastocyst formation,synaptonemal complex
ME17	intracellular membrane-bounded organelle,intracellular,intracellular transport
ME24	cell cycle,mitotic cell cycle process,mitotic cell cycle
ME12	RNA binding,nuclear lumen,nucleoplasm
ME1	sensory perception of chemical stimulus,G-protein coupled receptor activity,detection of chemical stimulus involved in sensory perception
ME52	mitochondrial genome maintenance,reproduction,vacuole inheritance
ME5	immune response,immune system process,leukocyte activation
ME37	detection of chemical stimulus involved in sensory perception,detection of chemical stimulus,detection of stimulus involved in sensory perception
ME55	detection of chemical stimulus involved in sensory perception of smell,olfactory receptor activity,sensory perception of smell
ME51	odorant binding,detection of chemical stimulus involved in sensory perception of smell,olfactory receptor activity
ME23	detection of chemical stimulus,detection of stimulus involved in sensory perception,detection of chemical stimulus involved in sensory perception of smell
ME30	myoblast fusion involved in skeletal muscle regeneration,interleukin-17E receptor binding,myotube differentiation involved in skeletal muscle regeneration
ME49	mitochondrial genome maintenance,reproduction,vacuole inheritance
ME33	animal organ development,keratinization,positive regulation of antibacterial peptide production
ME36	serotonin receptor signaling pathway,G-protein coupled serotonin receptor signaling pathway,G-protein coupled serotonin receptor activity
ME27	regulation of transcription from RNA polymerase II promoter involved in spinal cord association neuron specification,interleukin-20 binding,positive regulation of epithelial cell proliferation involved in wound healing
ME43	detection of chemical stimulus involved in sensory perception of smell,olfactory receptor activity,sensory perception of smell
ME19	detection of chemical stimulus involved in sensory perception of smell,olfactory receptor activity,keratinization
ME32	drug transport across blood-nerve barrier,cellular response to butyrate,regulation of steroid metabolic process
ME41	ectoderm development,GPI anchor biosynthetic process,GPI anchor metabolic process
ME45	peptide cross-linking,cornified envelope,keratinization
ME34	gonadal mesoderm development,spermatogenesis,male gamete generation
ME47	G-protein coupled receptor activity,G-protein coupled receptor signaling pathway,transmembrane signaling receptor activity
ME4	nuclear lumen,nucleoplasm,intracellular membrane-bounded organelle
ME6	intracellular membrane-bounded organelle,cytoplasm,intracellular organelle
ME53	mitochondrial genome maintenance,reproduction,vacuole inheritance
ME13	catalytic complex,mitochondrial envelope,intracellular membrane-bounded organelle
ME20	RNA binding,mitochondrion,intracellular membrane-bounded organelle
ME28	cytosolic ribosome,SRP-dependent cotranslational protein targeting to membrane,translational initiation
ME29	spliceosomal snRNP complex,mitochondrion,mRNA splicing, via spliceosome

Experimental GO enrichment identified for each nonTE module. After constructing a nonTE network, the modules were annotated using WGCNA. Please note that the annotations are experimental.

Supplementary table 2.

A.

Analysis of Deviance Table intra cohort TCGA validation (N=37)
Cox model: response is surv
Model 1: 14 TEP
Model 2: 14TEP+Age+Sex+Race
loglik Chisq Df P(>|Chi|)
1 -71.209
2 -71.274 0.1315 0.11354 0.12

B.

Pediatric AML (N=284)

Cox model: response is surv
Model 1: 14 TEP
Model 2: 14 TEP+Age+Sex+Race
loglik Chisq Df **P(>|Chi|)**
1 -666.03
2 -665.24 1.5854 1.7956 **0.4038**

C. Relapsed AML (N=19)

Cox model: response is surv
Model 1: 14 TEP
Model 2: 14 TEP+Age+Sex
loglik Chisq Df **P(>|Chi|)**
1 -35.488
2 -35.201 0.5735 0.023323 **0.01097 ***

Signif. codes: 0 '***' 0.001 '**' 0.01 '*' 0.05 '.' 0.1 ' ' 1

D.

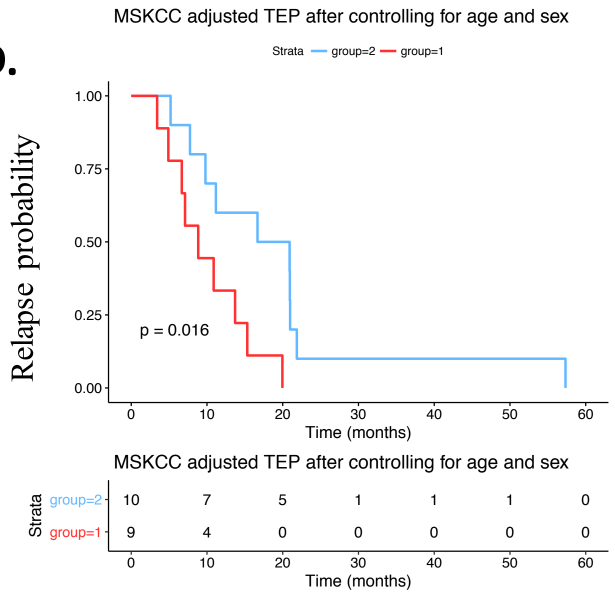

A. *Age, Sex, Race effects on 14 TEP in a Cox model.* In order to control for age, sex, and race, the demographical patient information was included with the 14 TEP as the covariates for a Cox PH model. This model was compared to the Cox model which included 14 TEP as the covariates. The demographical variables did not have an effect on the 14 TEP in the intra validation cohort (TCGA). **B.** *Age, Sex, and Race effects on TEP in pediatric AML.* The similar analysis was performed in pediatric AML. The demographical variables did not have an effect on TEP in pediatric AML. **C.** *Age and Sex effects on TEP in relapsed AML.* Age and sex were included with the 14 TEP as the covariates in a Cox model for relapsed AML. Age and Sex did have a significant effect on TEP in relapsed AML cohort. **D.** *KM model of adjust TEP hazard estimates (relapsed AML).* The age and sex adjusted TEP hazard estimates were used to formulate 2 relapse risk groups in relapsed AML.

Supplementary table 3.

AluSq2	*L1MB2*	*LTR24C*	*LTR56*	*MER11A*
IPO11	*ARHGEF5*	*SFSWAP*	*MPP4*	*CD79B*
LAPTM4B	*LZTS2*	*ASB2*	*SRSF4*	*NAV3*
PDE4C	*HECTD3*	*WNT6*	*FBXO15*	*ABCC9*
MTR	*MIA2*	*SFRP5*	*UBP1*	*SNU13*
RERG	*RNF25*	*TTLL2*	*PI4K2A*	*SIGLEC8*
ARHGEF4	*KCNV1*	*SARDH*	*PANK4*	*SFXN3*
DNAAF1	*C12orf50*	*TPTE2*	*LAPTM5*	*FBXW4*
FBXL18	*CHP2*	*CENPC*	*KCNV1*	*AKR1D1*
FBLIM1	*DAPK3*	*NTMT1*	*GLIPR1L2*	*SLC14A2*
STXBP4	*ZNHIT2*	*HTR3B*	*ZNF107*	*LACE1*
LOC100129931	*NPAP1*	*MIA2*	*PATE2*	*ARPC5L*
LOC101927740	*NHLRC1*	*PDE3B*	*ADAM32*	*GADL1*
NAV2-AS2	*LDLRAD2*	*FAM207A*	*MAP3K3*	*CXorf36*
S1PR2	*FAM180A*	*CLPB*	*FAM228B*	*HDAC11*
	WDR45	*DAPL1*	*LOC101928627*	*GABRR3*
	MIR1255A	*ZNF91*	*ERVMER61-1*	*RUSC2*
	LOC101927356	*MURC*	*LOC101926943*	*C17orf67*
	SLC39A12-AS1	*EIF4E1B*	*COX6CP14*	*LOC286370*
	LINC00276	*ZFP82*	*FOXP1-AS1*	*KIZ-AS1*
	LINC00458	*PHLDA2*	*LOC102546294*	*LINC01250*
	HSPE1P27	*IGSF5*	*GAS5-AS1*	*LOC102546299*
	TEX35	*GPR39*	*TSIX*	*LINC01541*
	LINC01011	*SERPINA11*	*PANK4*	*USP27X*
	LOC101928583	*ZNF124*	*C2-AS1*	*LOC158434*
	POU5F2	*CXorf40A*		
	RAD21-AS1	*SLC29A3*		
	SMIM18	*SPRED2*		
	PWRN1	*DHX16*		
	BIRC6-AS2	*SNORA15*		
		SNORD111		
		HCG25		
		HCG25		
		RNU6-625P		
		RPP21		
		RPP21		
		RNU6-678P		
		RNU6-703P		
		LINC00933		

AluJo	*MER44C*	*Tigger5A*
DYRK4	*FKBP6*	*BLOC1S6*
FGF13	*HUNK*	*ELF2*
HINT2	*NUDT13*	*CCDC90B*
MRPL49	*LOC729173*	*FAM126B*
VAC14-AS1	*SLC48A1*	*HTRA4*
TXNDC12-AS1	*LOC102546294*	*FAM169B*
FLJ33581	*MIR133A1HG*	*ZPBP2*
LOC284379		*PCDHA4*
C11orf98		*LOC101926943*
		LOC349160
		RAD21-AS1
		GHET1

TEP associated genes. The first entry at each column depicts various TEP. The entries underneath the column depict genes symbols that together predicted the corresponding TEP with an R^2 greater than 0.50. *MER31-I, LTR14A, MER101B, LTR45B,MER77, and Tigger9b* associated gene predictors had R^2 <0.5 and were not depicted.

Supplementary table 4.

A.

TCGA mixed effect model	TEP risk groups	cytogenetics	mutations	demographics	other	blood
coefficients	1.17651608	0.630706035	0.936094277	-0.545216442	-1.265302207	0.467890025
exp(coef)	3.243055951	1.878936706	2.550002341	0.579716295	0.282154014	1.596621805
variance.random.effects	0.000396111	0.000396425	0.000396147	0.000393576	0.000396263	1.140686347
p.value.mixed.effects.model	0.054	0.023	0.0017	0.16	0.049	0.41
std.deviation.effects.model	0.0199	0.01991	0.0199	0.01984	0.01991	1.068

A. *TCGA intra cohort validation multivariate Cox PH model with random effects for covariate group.* The rows correspond to statistics resultant from a multivariate model which included mixed effects per covariate group. Each individual covariate was summarized into a categorical group feature. The columns correspond to the variables included in the model. The '*other*' covariate group included transplantation status (allo, auto, MUD).

B.

TCGA	TE nested model log-likelihood	model (TE, cytogenetics, mutations, demographics, clinical and blood) log	TE.vs.fullModel.pvalue	Chisq	DF
ANOVA	-48.88085045	-55.88220865	0.232843204	14.003	11

B. *ANOVA comparing multivariate Cox PH model with random effects to the nested Cox PH model of TEP risk groups using all TCGA subjects (N=178).* The ANOVA compared the multivariate model, similar to ***A***, to the nested Cox PH model that included only TEP risk groups. Similarly, the intra-validation ANOVA resulted in a p.value of 0.962.

C.

TARGET mixed effect model	TEP risk groups	cytogenetics	mutations	demographics	other
coefficients	0.371233118	-0.019826321	-0.53396977	-0.100382637	0.118761897
exp(coef)	1.449520944	0.980368928	0.586272975	0.90449126	1.126101758
variance.random.effects	1.19E-09	4.87E-11	6.39E-07	4.59E-05	0.000145665
p.value	0.036	0.85	0.0032	0.31	0.1
std.deviation.mixed.effects	3.45E-05	6.98E-06	0.0007993	0.0067741	0.012069

C. *(Pediatric AML) Multivariate Cox PH model with random effects for covariate group.* Similar analysis to ***A***. Each individual covariate was summarized into a categorical group. The '*other*' covariate group included minimum residual of disease (MRD) at the end of course 1 treatment, stem cell transplant in first complete remission, MRD at the end of course 2, white blood count at diagnosis, bone marrow blast percentage, and peripheral blood blast percentage.

D.

TARGET	TE nested model log-likelihood	full model (TE,cytogen, mutations, demographics, clinical) log-likelihood	TE.vs.fullModel.pvalue	Chisq	DF
ANOVA	-676.4461793	-682.1008178	0.255103813	11.309	9

D. *ANOVA comparing multivariate Cox PH model with random effects to the nested Cox PH model of TEP risk groups.* The ANOVA compared the multivariate model, from ***C***, to the nested Cox PH model that included only TEP risk groups.

E.

MSKCC mixed effect model	TEP risk group	cytogenetics	mutations	demographics	white blood count
coefficients	-1.923872645	1.126241504	0.42015834	0.148316122	-0.176006472
exp(coef)	0.146040303	3.084043327	1.522202562	1.159879502	0.838612556
variance.random.effects	0.000399547	0.000399493	0.000399622	0.000399307	0.000399349
p.value	0.014	0.046	0.4	0.74	0.77
std.deviation.random.effects	0.019989	0.019987	0.01991	0.019983	0.019984

E. *(Relapsed AML) Multivariate Cox PH model with random effects for covariate group.* Similar analysis to ***A***.

F.

MSKCC	TE nested model log-likelihood	full model (TE,cytogen, mutations, demographics, clinical) log-likelihood	TE.vs.fullModel.pvalue	Chisq	DF
ANOVA	-34.9814353	-37.74022365	0.787060237	5.176	9

F. *ANOVA comparing multivariate Cox PH model with random effects to the nested Cox PH model of TEP risk groups in relapsed AML.* The ANOVA compared the multivariate model, from ***E***, to the nested Cox PH model that included only TEP risk groups.

Methods:

Acquisition of RNA sequencing fastq files for AML

The RNAseq data was obtained from The Cancer Genome Atlas (TCGA) via dbGAP access. The transcriptome data for the 284 pediatric AML (TARGET[1]) and relapsed AML[2] were also obtained through dbGAP.

Normalization of TE and non-TE in TCGA

We calculated transcript abundances using Kallisto[3], which estimated transcript abundances against a comprehensive catalog of transcriptomic data consisting of ERCC (Ambion) Spike-Ins version 97, transposable element (TE) transcriptome annotations derived from RepBase GIRI release 2005 for Homo-Sapiens, and ENSEMBL non-TE (non-TE) Annotations build 80. The expression data was compiled into a "kallisto experiment" using *Arkas*[4], which included annotation information, TE transcript identifications, TE transcript biotype and TE biotype classes of TE and non-TE class information.

We considered normalized expression of TE and non-TE by collapsing all read counts with a minimum read cutoff of 16, as recommended the sequencing quality control consortium (SEQC)[21]. The non-TE and TE were normalized together as a source data using voom[5] and a design matrix that included gender, race, age, and 5% prevalent mutations affecting protein changes (FLT3, NPM1, DNMT3A, IDH1/2, RUNX1, TET2, TP53, NRAS, KRAS, CEBPA, WT1, PTPN11, KIT, MT.CO2, TTN, U2AF1, SMC1A, SMC3, and STAG2), cytogenetics (PML-RARα, MLL.partner, inv(16), RUNX1.RUNX1T1, MLLT10.partner, MLL partial tandem duplication, deletion 5/5q, delection 7/7q, delection 11.11q23, deletion 20q, deletion Y

chromosome, any abnormality on chromosome 17, trisomy 8, and trisomy 21). Additional features included were ‘blood’ variables defined as bone marrow blast percentage, peripheral blood blast percentage and white blood counts at diagnosis. Transplantation profile for each patient such as allogeneic donor transplantation, auto-stem cell transplantation, or matched unrelated donor transplantation were included into the model. The voom algorithm corrects heteroscedasticity, and the normalized counts were then saved in $\log_2$ counts per million (CPM).

Normalization of TE and non-TE in TARGET

The cytogenetic variables included in the pediatric TARGET patient profile were t(8;21), inv(16), deletion 7q, deletion 9q, trisomy 8, trisomy 21, Y deletion, X deletion, t(9;11), t(10;11), and t(11;19). The mutations included were FLT3 ITD positive, FLT3 point mutation, NPM point mutation, CEBPA mutation, WT1 mutation, and KIT mutation on exon 17 and 8. Minimal residual of disease status at the end of 1st, 2nd course of treatments were identified for each patient, along with stem cell transplantation status. Gender, race, and age at diagnosis were additional factors included in the patient profile. White cell count at diagnosis, bone marrow blast percentages, and peripheral blood blast percentages were also included. The patient profiles with the listed features were used to normalize the non-TE and TE expression data using 'voom'.

Normalization of TE and non-TE in MSKCC

For the 19 relapsed AML patient profiles, the following predictive features were used in normalizing nonTE/TE expression and survival analysis. Demographic variables were age, and sex. Mutational variables were NPM1 point mutations, FLT3 point mutations. Cytogenetic

variables were deletion 5 on the q-arm, deletion of 7 on the q-arm, Y deletion, trisomy 21, t(6;9), and inversion 16. Other variable white blood counts were also used.

Multivariate linear model: altered expression of transposable elements in TCGA

We fit the normalized TE expression in an empirical Bayesian linear model and identified altered expressed TE (using TE transcripts), using Benjamini-Hochberg FDR cutoff of 0.05, un-adjusted p.value threshold of 0.01, minimum absolute log-fold change of 0.5, and hierarchical multiple testing strategy across features as contrasts[6].

The altered expressed TE (AE-TE) were then identified which satisfied the previously described filtering criteria for each corresponding feature such as mutations, cytogenetics, blood related factors, and clinical/demographic factors resulting in significant effects per feature. The total numbers of AE-TE were plotted in a barplot for each predictor variable. We compared the linear model to a random model by sampling randomly the predictor design matrix and re-fitting an empirical Bayesian random mutational model[7].

The statistically significantly AE-TE identified after hierarchical testing were summarized by averaging the estimated coefficients for each corresponding AE-TE within each biotype. In other words, for each TE biotype, the significant AE-TE estimated coefficients from the linear model were averaged. The biotype information was identified using *Arkas*[4], which includes RepBase annotation information at the transcript level. We hence were able to identify each TE transcript biotype dysregulation for each mutational predictor. Each of these was depicted using an R package ‘riverplot’.

Multivariate linear model: altered expression of ENSEMBL non-TE TCGA

Normalized transcripts of non-TE were examined separately from TE. Non-TE were identified using ENSEMBL gene identifiers, which included coding gene, pseudogene, long noncoding RNA, short noncoding RNA elements, and small nuclear RNA. *Arkas* provided the annotation information and data stored of the experimental data. The multivariate linear model, and significant effects were similarly modeled as with TE; we used hierarchical filtering of the multivariate model coupled with Benjamini-Hochberg FDR filtering q.value threshold of 0.05, un-adjusted p-value 0.01 threshold, and minimum absolute log fold change threshold of 0.5.

Coding gene network association table with TE transcript biotypes

In order to comprehensively study the correlation patterns between TE and other transcripts including coding gene pathways, we constructed a weighted gene correlation network table[8]. We first constructed a non-TE expression network using WGCNA using soft-thresholding power of 6 after confirming that scale free topology and mean-connectivity scores were above 80%, which suffices for diverse nodal connectivity. The normalized non-TE transcript expression in $\log_2$ CPM was used for ENSEMBL gene-IDs described earlier. The minimum genes for each eigen-gene cluster was set to 10, the maximum was set to 4000. We used ‘blockwise’ module construction, and module 50 is defined as the set of genes that remained un-connected in the networking context.

Each gene module was then correlated to the normalized expression of the TE biotypes selected as Alu, hAT,TcMar, Mariner/Tc1, PiggyBac, L1, Pseudogene, MULE, KER, Gypsy, CR1, RTE, centromeric, Satellite, Eutr10, SVA, MSAT, Transposable Element, MIR, L2,

HSFAU, ERVK, SINE2-tRNA, Eutr11, ERV3, ERVL, Repetitive Element, SAT, DNA Transposon, Endogenous Retrovirus, Merlin, Retrotransposon, telo, snRNA, satellite, acromeric, and ERV1. Each of these biotypes was identified using RepBase as previously described using *Arkas*. The correlation values were computed by correlating the compiled TE biotype expression, which was obtained by summing normalized corresponding TE transcripts belonging to the particular biotype, to the module eigengene first principal component[8, 9]. Statistically significant correlations of biotypes to gene networks used a pearson correlation test with a significance threshold of 0.05.

The module average dysregulation for multiple predictive features was determined using the multivariate linear model of non-TE as previously described. After hierarchical FDR filtering of the non-TE, the AE non-TE per each module was then identified. The module summary dysregulation was defined as the mean of the empirical Bayesian estimated coefficients from the corresponding statistically significant AE-non-TE within each module (Supplementary figure 2).

<u>Prognostication of AML using TE transcript expression in TCGA:</u>

We used all patients in TCGA to normalize. We prepared the TE expression of all patients by normalizing the collapsed estimated counts with respect to ENSEMBL transcript IDs using limma-voom[11] as previously described. We then subset the training and testing cohort after normalization. The corresponding survival times along with expiration status of each patient was obtained from the data acquisition. The matching survival data for each data split was matched by patient.

Identification of 14 transposable element prognosticators via nested sampling

We identified the 14 candidate transposable element prognosticators through nested sampling in TCGA (N=178). For each nested sample division, the TCGA subjects were divided 60% nested training cohort, and 40% nested testing cohort. Each nested cohort division ensured that selected covariates were prevalent within the subdivision proportional to the population prevalence of the corresponding covariate. The following covariates were frequency matched: *TP53, FLT3, NPM1, DNMT3A, FLT3/NPM1* mutation combinations, *FLT3/DNMT3A* combinations, *PML-RAR*α, peripheral blood blast % and a broad cytogenetic category defined as ‘Other in frame fusions’. The broad category ‘Other in frame fusions’ included fusions such as *FLT3LG-RPS11, C11orf1-KIAA0999, BIRC6-LTBP1, CREBBP-KAT6A, CRID1-DPM1, RUNX1-MECOM, RPN1-MECOM, GOSR1-ZNF207, NF1-RRC37B, DDX20-TBX15,* and *CHST11-ATP1B4.* Each cohort division used the population prevalence of the given mutation/cytogenetic/etc. to allocate subjects into each cohort ensuring the subset cohort prevalence for the given feature was balanced with respect to gender and proportional to the population prevalence. The allocation of subjects was performed using random sampling, ensuring that each division had balanced genders on selected clinically important covariates. The following nested sampling process was used:

1. The outer training samples N_i (60%) and outer-validation samples V_i (40%) are identified using frequency matching on gender and population prevalence as previously described. Let the outer iteration be the ith index, and the nested inner iteration be defined as the kth index.

2. The training samples N_i are divided into nested training set (50%) S_{ik} and nested validation set (50%) T_{ik}. Given the nested training set S_{ik}, univariate feature selection[13] based on univariate significance Wald test with significance threshold of 0.0125 and a 3-fold cross validation is performed. The set of univariate selected features identified from S_{ik} is then modeled into a Cox multivariate proportional hazards model using nested validation set on the ith outer fold and k^{th} inner fold, T_{ik}. The concordance index from the inner validation is calculated and the set of univariate selected features would be discarded if the C-statistic was below 0.6.
3. The nested sampling of N_i was iterated 100 times (k:1-100). Each nested iteration returned a set of univariate selected features whose multivariate c-index was observed in the corresponding nested validation to be at least 0.60.
4. The total frequency of each transposable element selected in nested sampling of N_i **(3)** was tabulated. The TEs which were frequently re-observed across nested re-sampling on the ith fold (at least in the 90th percentile) were selected as the candidate TEP_i for the i^{ith} fold.
5. The candidate TEP_i were then validated in the ith outer fold V_i (40%). The candidate TEP_i were evaluated using the concordance index and the Wald test statistic from a Kaplan-Meier (KM) survival plot. The validation cohort V_i was classified into 2 risk groups derived from compound covariate prediction method[12,13,15]. The compound covariate prediction method is based on a linear combination of the univariate Cox regression estimates and the corresponding expression scores of the predictor[12-14]. The 2 risk groups were modeled into a KM survival plot and the Wald test was performed.

6. The steps 1-5 were iterated 5 times which returned a final set of 5 candidate TEP sets for each iteration (e.g. {TEP_1, …, TEP_5}. The final set of candidate TEP were identified by selecting TE frequently observed across the set of candidate TEPs. TEPs with frequencies at least in the 75th percentile across each of the 5 iterations were the final candidates.
7. The intra cohort validation test was performed by sampling approximately 20% of TCGA (N=37). The final set **TEP** were validated using the intra-test validation set and depicted in figure 3A.

Marcucci et.al implemented a univariate Cox model to identify potential prognostic miRNAs[14]. We utilized a similar univariate Cox proportional hazards regression model with stringent alpha levels to identify subsets of covariates[12]. We identified 14 Transposable Element Prognosticators (**TEP**) in TCGA defined as *AluJo, AluSq2, L1MB2, LTR14A, LTR24C, LTR56, MER101B, MER11A, MER31-I, MER44C, Tigger5A, LTR45B, MER77, Tigger9b.*

Evaluation of TEP

The survival analysis was evaluated in three ways.

1. We implemented multivariate Cox regression using a ridge regression[13] and the C-statistic was evaluated using cross-fold validation. For TCGA intra cohort validation, we used 3-fold cross validation (N=37).
2. Utilizing compound covariate prediction method, (identifying patient risk scores as a linear combination of **TEP** expression and **TEP** hazard estimates), the patient risk summary scores were cut into 2 risk groups using a quantile cut at the 75th quartile. A

Kaplan-Meier (KM) survival model was performed using the 2 groups, and a Wald test statistic was calculated on the classification of patient risk summary scores.

Simple sign averaging method for risk discrimination

Simple sign averaging of Cox hazard estimates was shown to perform no worse than LASSO, or ridge regression[15]. We calculated the sign average estimated from the TCGA intra cohort validation using the final **TEP.** Using the sign averages of the Cox hazard estimates, we perform a Kaplan-Meier (KM) survival analysis on the intra cohort validation set (N=37), TARGET, and MSKCC.

Cox proportional hazards model with random biotype effects TCGA

The **TEP** were fit into a multivariate Cox model with random effects which assumed a Gaussian distribution for the random effects[16] (Therneau 2012,, https://doi.org/10.1198/1061860031365, A. Perperoglou (2014). Cox models with dynamic ridge penalties on time-varying effects of the covariates. Stat Med, 33:170-80. http://dx.doi.org/10.1002/sim.). The **TEP** biotypes were modeled using the package 'CoxRFX', which computed a random effects model assuming that each **TEP** biotype shared a normal distribution. (Supplementary figure 3C). The **TEP** constituted 2 Alus, 5 ERV1s, 2 ERKs, 3 TcMars, 1 ERVL, and 1 L1. The **TEP** Cox model with random effects per biotype did not include ERVL nor L1 into this model because these biotypes were sparse.

Cox proportional hazards model using covariates such as 14 TEP, Age, Sex and Race TCGA

In order to control for potential demographical confounders such as age, sex and race, they were included into the Cox model[15] that included 14 TEP covariates. The analysis of deviance (ANOVA) was performed comparing the nested Cox model that included 14 TEP, to the model that used 14 TEP, age, sex and race. The intra cohort validation in TCGA was examined, and the full TCGA model was examined.

Cox multivariate hazards model with random effects for group categorical covariates in TCGA

TCGA intra cohort validation of **TEP** using multivariate Cox proportional hazards model with random effects for group covariates was assessed. The package 'coxme' was used to calculate the statistics of a multivariate Cox model that includes random effects for each group covariate. The group covariates included were TEP risk groups (Figure 3B), cytogenetics, mutations demographics, other, and blood. The TEP risk groups was determined from the TEP hazard estimates calculated from a Cox model[15]; the subsequent patient risk scores were used to classify patients into 2 risk categories. Note that Supplementary table 2 used the TEP signed averages as the discriminators used for patient risk group classification that derived 'TEP risk groups'. Hence the variable 'TEP risk groups' was a categorical variable. The variable 'cytogenetics' was defined as the sum of all cytogenetic covariates (indicator variables) for each patient in the cohort. The 'mutations', 'demographics', 'other' (which was the transplantation status), and 'blood' covariates were tabulated similarly. The ANOVA was performed comparing the multivariate model to the nested model of only the 'TEP risk groups'.

Cox proportional hazards multivariate model risk contributions in TCGA

The multivariate Cox model included covariates such as nonTE, TEP, cytogenetics, mutations, blood, demographics, and other. The nonTE covariates were derived using the first 14 principal components (PC) of all nonTE expression[15]. The 14 nonTE PCs were mapped into a binary variable by cutting the values on the 75th quartile.

The 14 TEP covariates were the PCs of the expression of the 14 TEP and the components were cut on the 75th quartile. The 'cytogenetic' covariates used in this Cox model were *PML-RARα*, *MLL*.partner, inv(16), *RUNX1.RUNX1T1*, *MLLT10*.partner, *MLL* partial tandem duplication, deletion 5/5q, delection 7/7q, delection 11.11q23, deletion 20q, deletion Y chromosome, any abnormality on chromosome 17, trisomy 8, and trisomy 21. The 'mutation' covariates were *FLT3, NPM1, DNMT3A, IDH1/2, RUNX1, TET2, TP53, NRAS, KRAS, CEBPA, WT1, PTPN11, KIT, MT.CO2, TTN, U2AF1, SMC1A, SMC3,* and *STAG2*. The covariate 'other' was allo, auto, MUD transplant status. The 'demographic' covariates were sex, age, ethnicity. The 'blood' features were BM/PB blast %, and WBC. All variables were then mean centered and fit into a Cox proportional hazards model, each variable's contribution to the multivariate concordance index is depicted in Figure 6. The survival plots were drawn using the R package 'survminer' (Alboukadel, 2017, https://CRAN.R-project.org/package=survminer).

Validation in TARGET Pediatric AML

We validated the 14 TEP expression using 284 pediatric AML patients. The expression data was normalized using limma-voom. The design matrix considered mutation and cytogenetic information that was 5% prevalent in the TARGET cohort. The normalized **TEP** expression were fit into a Cox model[15] and the hazards estimates were used to identify patient risk scores as

previously described. The patient risk summary scores classified patients into 2 groups based on a quantile cut at the 75th quartile. The patient risk group classification was then fit into a KM survival plot depicted in Figure 3C.

The signed averages of the TARGET **TEP** hazard estimates were used as an alternative discrimination. The signed averages were computed as previously described and depicted in Supplementary figure 4. The signed averages were used to derive patient risk summary scores, and subsequently used to classify patients into 2 risk groups. These risk groups were fit into another KM survival model and the Wald test statistics were calculated (Supplementary figure 4 C and D). The C-statistic was calculated using 5-fold cross validation from the Cox model[15].

The effects of age, sex, and race covariates were examined. The ANOVA was performed on a Cox model with covariates such as 14 TEP, age, sex and race, to the nested Cox model with 14 TEP. The table is listed in supplementary table 2B.

Cox multivariate hazards model with random effects for group categorical covariates in TARGET

The multivariate Cox model with clinically relevant features along with the TEP in TARGET was assessed. As previously described, the 'coxme' package was used to model group level covariates along with random effects per group in a Cox model. The covariates were derived by tabulating the indicator variables. The 'TEP risk groups' variable was derived using signed averages, which tended to be more conservative (Supplementary table 3 C and D).

Validation in Adult Relapsed AML Memorial Sloan Kettering Cancer Center (MSKCC) data set

We normalized the expression of MSKCC 19 relapsed AML patients using voom. The 14 TEP normalized expression was similarly used in a Cox model[15], and the patient risk classification groups were identified. The KM survival model was constructed, and depicted in Figure 3D. The Wald test statistic was computed from the KM model and depicted in supplementary figure 4F.

The effects of age, and sex covariates were examined in MSKCC. The patient ethnicity information was not available. The ANOVA was performed similar to TARGET and TCGA. The table listed in supplementary table 2C shows that there are significant effects on TEP from age and sex in relapsed AML cohort. The adjusted TEP hazard rates were derived from a Cox model that included 14 TEP covariates along with age and sex. The sign averages of the adjusted TEP hazard estimates were compounded with the corresponding TEP expression which identified patient risk summary scores. These adjusted risk scores were cut on the 75th quartile to identify 2 patient risk groups that were modeled in a KM survival model (supplementary table 2D). The Wald test statistics were computed on the KM model.

Cox multivariate hazards model with random effects for group categorical covariates in MSKCC

The risk group classification categorical covariates were fit into a multivariate Cox model with random effects per group. Cytogenetics, mutations, demographics, and white blood count (WBC) were summarized into a group categorical covariate using ‘coxme’.

TCGA stratified survival models based on mutational covariates

Using mutation status of 178 patients and their corresponding event free survival times (EFS), we predicted survival using Cox model variant that used ridge penalty[15]. The mutational status in the Cox model variant were centered, but not scaled[13]. Survival statistical testing was performed consistently as before. We thus identified 96 low risk patients determined by mutation covariates, and 82 patients as high risk based on mutations alone.

Using the 14 TEP and the 96 low-risk patients classified by mutations, we performed survival analysis using Cox model to sub-stratify the low-risk mutation based cohort. The TEP expression of the mutation based low-risk patient cohort was transformed into a categorical variable. The principal components of the 14 TEP expression[15] corresponding to the patients within the low-risk cohort were cut at the 75th quartile. The categorical TEP features were centered and hazards were estimated from a Cox model. This model was evaluated in a Kaplan-Meier survival model as previously described. The summary statistics, and statistical testing of the survival analysis was performed consistently as before. Although dichotomizing the continuous TEP covariates is not necessary, we attempted to negatively bias the TEP’s predictive power for a conservative analysis.

High risk (N=82) patients classified by mutations were also sub-stratified. The TEP sub-stratification was performed identically as the low-risk analysis.

TCGA stratified survival models based on cytogenetic covariates

Similar to mutational risk classifications, we identified risk groups by compounding the Cox model hazard estimates derived from cytogenetics previously described. Each cytogenetic risk cohort was analyzed similarly to the mutation sub-stratification analysis using the 14 TEP.

TCGA stratified survival models based on nonTE expression covariates

The nonTE expression of the 178 TCGA patients were used to identify patient risk groups. The 'voom' normalized expression was performed, and the principal components were identified. The first 14 PCs were used as covariates in the Cox model[15] and were cut into a categorical variable using the 75th quartile. These categorical features were centered, and then used in a Cox model and the concordance index was computed using a 3-fold cross validation. The nonTE risk groups were identified using the resultant hazard estimates that were compounded with the categorical non-TE PCs variables.

Similar to the identification of risk cohorts using the nonTE expression, the principal components of the TEP were similarly utilized. The nonTE based low-risk patient cohort was used as the subjects for sub-stratification analysis. The principal components of the subjects' corresponding 14 TEP expression were dichotomized into a categorical variable by cutting at the PCs 75th quartile. These categorical variables were centered and used as the features in the Cox model which identified hazard estimates per each categorical variable. Compounding the Cox

covariates with the estimated hazards determined 2 sub-stratified risk groups. These 2 risk groups were used in a Kaplan-Meier survival model (Figure 4E).

The high-risk cohort identified by nonTE expression was modeled similarly (Figure 4F).

TEP associated gene predictors identified through penalized regression

The normalized nonTE expression was used as the independent predictors for each of the 14 TEP (response variable) using 'glmnet'[18]. The LASSO (Gaussian family) was used with 10 fold cross-validation (10). The largest 'lambda' within 1 error of the minimum cross validated error was used. The scaled predicted response was derived from the glmnet model which was compared to the scaled observed TEP expression. Figure 5 depicted the gene predictors which modeled the corresponding TEP expression (R^2 >0.5).

Previously the nonTE network identified module membership for each nonTE. The module membership for the selected TEP predictors was identified using the nonTE network (Figure 2).

Functional annotation of TEP associated predictors

The DAVID[19] bioinformatic resource was used to identify GO function annotations associated with genes from module 2 and 3. We screened the enriched GO functions using an FDR cutoff of 0.05 and a Fisher p.value threshold of 0.05. GO biological processes (BP), molecular function (MF), and cellular components (CC) were identified for module 2 and 3.

References:

1. Hamid Bolouri, Jason E. Farrar, Timothy J. Triche Jr., Rhonda E. Ries, Emilia L. Lim, Todd A. Alonzo, Yussanne Ma, Richard Moore, Andrew Mungall, Marco A. Marra, Jinghui Zhang, Xiaotu Ma, Yu Liu, Yanling Liu, Jaime M. Guidry Auvil, Tanja M. Davidsen, Patee Gesuwan, Leandro C. Hermida, Bodour Salhia, Stephen Capone, Giridharan Ramsingh, Christian Michel Zwaan, Sanne Noort, Stephen Piccolo, E. Anders Kolb, Alan S. Gamis, Malcolm A. Smith, Daniela S. Gerhard, Soheil Meshinchi. Comprehensive characterization of pediatric AML reveals diverse fusion oncoproteins and age-specific mutational interactions. *BioRxiv*, bioRxiv 125609; doi: https://doi.org/10.1101/125609 (2017).

2. Li, S. *et al*. Distinct evolution and dynamics of epigenetic and genetic heterogeneity in acute myeloid leukemia. *Nat. Med.* **22**, 792-799 (2016).

3. Bray, N. L., Pimentel, H., Melsted, P. & Pachter, L. Near-optimal probabilistic RNA-seq quantification. *Nat. Biotechnol.* **34**, 525-527 (2016).

4. Colombo, A. R., J Triche, T.,Jr & Ramsingh, G. Arkas: Rapid reproducible RNAseq analysis. *F1000Res* **6**, 586 (2017).

5. Law, C. W., Chen, Y., Shi, W. & Smyth, G. K. voom: Precision weights unlock linear model analysis tools for RNA-seq read counts. *Genome Biol.* **15**, R29-2014-15-2-r29 (2014).

6. Ritchie, M. E. *et al*. limma powers differential expression analyses for RNA-sequencing and microarray studies. *Nucleic Acids Res.* **43**, e47 (2015).

7. Gerstung, M. *et al*. Combining gene mutation with gene expression data improves outcome prediction in myelodysplastic syndromes. *Nat. Commun.* **6**, 5901 (2015).

8. Langfelder, P. & Horvath, S. WGCNA: an R package for weighted correlation network analysis. *BMC Bioinformatics* **9**, 559-2105-9-559 (2008).

9. Langfelder, P. & Horvath, S. Fast R Functions for Robust Correlations and Hierarchical Clustering. *J. Stat. Softw* **46**, i11 (2012).

10. Yaari, G., Bolen, C. R., Thakar, J. & Kleinstein, S. H. Quantitative set analysis for gene expression: a method to quantify gene set differential expression including gene-gene correlations. *Nucleic Acids Res.* **41**, e170 (2013).

11. Robinson, M. D., McCarthy, D. J. & Smyth, G. K. edgeR: a Bioconductor package for differential expression analysis of digital gene expression data. *Bioinformatics* **26**, 139-140 (2010).

12. Emura, T., Chen, Y. H. & Chen, H. Y. Survival prediction based on compound covariate under Cox proportional hazard models. *PLoS One* **7**, e47627 (2012).

13. Gerstung, M. *et al*. Combining gene mutation with gene expression data improves outcome prediction in myelodysplastic syndromes. *Nat. Commun.* **6**, 5901 (2015).

14. Marcucci, G. *et al*. MicroRNA expression in cytogenetically normal acute myeloid leukemia. *N. Engl. J. Med.* **358**, 1919-1928 (2008).

15. Zhao, S. D., Parmigiani, G., Huttenhower, C. & Waldron, L. Mas-o-menos: a simple sign averaging method for discrimination in genomic data analysis. *Bioinformatics* **30**, 3062-3069 (2014).

16. Ripatti, S. & Palmgren, J. Estimation of multivariate frailty models using penalized partial likelihood. *Biometrics* **56**, 1016-1022 (2000).

17. Parker, H. S. *et al*. Preserving biological heterogeneity with a permuted surrogate variable analysis for genomics batch correction. *Bioinformatics* **30**, 2757-2763 (2014).

18. Friedman, J., Hastie, T. & Tibshirani, R. Regularization Paths for Generalized Linear Models via Coordinate Descent. *J. Stat. Softw* **33**, 1-22 (2010).

19. Huang da, W., Sherman, B. T. & Lempicki, R. A. Systematic and integrative analysis of large gene lists using DAVID bioinformatics resources. *Nat. Protoc.* **4**, 44-57 (2009).

20. Gu, Z., Eils, R. & Schlesner, M. Complex heatmaps reveal patterns and correlations in multidimensional genomic data. *Bioinformatics* **32**, 2847-2849 (2016).

21. SEQC/MAQC-III Consortium. A comprehensive assessment of RNA-seq accuracy, reproducibility and information content by the Sequencing Quality Control Consortium. Nat Biotechnol. 2014 Sep;32(9):903-14. doi: 10.1038/nbt.2957. Epub 2014 Aug 24.
